# Islands and Non-islands in Native and Heritage Korean

**DOI:** 10.3389/fpsyg.2016.00134

**Published:** 2016-02-15

**Authors:** Boyoung Kim, Grant Goodall

**Affiliations:** ^1^Department of Asian Studies, University of Texas at AustinAustin, TX, USA; ^2^Department of Linguistics, University of CaliforniaSan Diego, La Jolla, CA, USA

**Keywords:** island constraints, Korean, heritage speakers, acquisition, scope ambiguity, *wh-in-situ*

## Abstract

To a large extent, island phenomena are cross-linguistically invariable, but English and Korean present some striking differences in this domain. English has *wh*-movement and Korean does not, and while both languages show sensitivity to *wh*-islands, only English has island effects for adjunct clauses. Given this complex set of differences, one might expect Korean/English bilinguals, and especially heritage Korean speakers (i.e., early bilinguals whose L2 became their dominant language during childhood) to be different from native speakers, since heritage speakers have had more limited exposure to Korean, may have had incomplete acquisition and/or attrition, and may show significant transfer effects from the L2. Here we examine islands in heritage speakers of Korean in the U.S. Through a series of four formal acceptability experiments comparing these heritage speakers with native speakers residing in Korea, we show that the two groups are remarkably similar. Both show clear evidence for *wh*-islands and an equally clear lack of adjunct island effects. Given the very different linguistic environment that the heritage speakers have had since early childhood, this result lends support to the idea that island phenomena are largely immune to environmental influences and stem from deeper properties of the processor and/or grammar. Similarly, it casts some doubt on recent proposals that islands are learned from the input.

## Introduction

A well-known fact about filler-gap dependencies in natural language is that gaps are not allowed in certain structural environments, known as islands. Interrogative clauses (*wh*-clauses or whether-clauses) are one such environment, for instance, as seen in (1).

(1) a. *Who do you wonder [why Mary saw __ ] ?
b. *Who do you wonder [whether Mary saw __ ] ?

One interesting fact about islands is that to a very large extent, they are cross-linguistically invariable. That is, environments where gaps are disallowed in English often have this same characteristic in other languages. Likely related to this is the fact that children's sensitivity to islands does not seem to depend in any obvious way on their being exposed to direct evidence for them. Children clearly hear evidence for filler-gap dependencies and for structures such as *wh*-clauses, for instance, but it is not clear if anything in the environment would suggest to children that gaps should not be allowed within such clauses. For this reason, many have suggested that islands are not learned directly, but instead follow from constraints on processing ability (e.g., Kluender, 1998; Hofmeister and Sag, 2010) or grammar (e.g., Chomsky, 1971; Rizzi, 2013). In recent years, however, some have suggested that initial appearances notwithstanding, islands are in fact able to be learned from the environment (e.g., Culicover and Jackendoff, 2005; Pearl and Sprouse, 2013). Under this type of approach, children use statistical mechanisms to track, analyze, and generalize patterns in the input. On the basis of the generalizations attained, they are able to produce and comprehend sentences beyond their experience, while prohibiting patterns not warranted by their experience.

Despite the apparent cross-linguistic uniformity of islands, there is nonetheless some variability. To begin with, many languages do not have overt *wh*-extraction, adopting instead a wh *in-situ* strategy, in which the *wh*-phrase occupies what would otherwise be the gap position. In such languages, there is thus no overt filler-gap dependency. This may be seen in the Korean example in (2), in which the *wh*-phrase nwukwu-ul “who” is located in the embedded clause, and the scope of this phrase is indicated by the question particle –ni in the matrix clause.

(2) Mary -nun [Obama-ka nwukwu-ul manna-ss-ta-ko]
-Top -Nom                  who -Acc   meet-Past-Decl-Comp
malhae-ss-ni?
say -Past-Q
             “Who did Mary say that Obama met ___?”

As will be discussed below, the *wh*-phrase here may be also interpreted as an existential pronoun, in which case the question particle –ni would signal a yes/no question, but the important point here is that even under the *wh*-question interpretation, nwukwu-ul remains *in-situ*.

Even in languages of this type, though, it has sometimes been claimed that islands are still obeyed, in the sense that the *in-situ wh*-phrase is degraded when inside an island structure. This has been claimed for interrogative clauses in Japanese and Korean, for example, as seen in (3) for Korean (claimed to be unacceptable under the *wh*-question reading given here).

(3) *Mary –nun [Obama-ka nwukwu-ul manna-ss-nun-ci]
-Top –Nom                    who -Acc   meet-Past-And-Q
ahl-ass-ni?
know-Past-Q
     “Who did Mary know whether Obama met ___?”

The picture is not quite this simple, though, since some environments that appear to be islands in *wh*-movement languages nonetheless allow *wh*-phrases in wh *in-situ* languages. Adjunct clauses provide an example of this. (4) shows that they are typically islands in a language like English, while (5) shows that they appear to allow *wh*-phrases in a language like Korean.

(4) *Who did Mary appear [when Obama met __ ]?

(5) Mary-nun [Obama-ka **nwukwu**-ul   manna-ss-ul-**ttay**]
-Top -Nom who -Acc                      meet-Past-Adn-when
natana-ss-**ni**?
appear-Past-Q
           “Who did Mary appear when Obama met?”

What we have seen so far describes the knowledge of monolingual speakers of languages like English and Korean. Bilingual speakers of two languages with these properties would very reasonably be expected to be different. Bilinguals receive less input for each of their languages than do monolinguals for their single language. For heritage speakers in particular (i.e., early bilinguals who grew up with exposure to the heritage language (L1) and the majority language (L2) either simultaneously or sequentially in early childhood, but whose L2 became the primary language at some point during childhood), there are additional factors. Their exposure to the heritage language in childhood may have been limited in important ways, their acquisition of the heritage language may have been incomplete, and they may have undergone significant attrition in the years since childhood (e.g., Anderson, 1999; Montrul, 2002; Sorace, 2004; de Groot, 2005; Polinsky, 2011a). In addition, for all bilinguals, there is the possibility of transfer, that is, that properties of one language will influence the other. For bilinguals in general, and heritage speakers in particular, it is well known that these environmental differences can lead to very significant differences between them and native speakers (e.g., Polinsky, 2011b and references cited there).

Bilinguals thus present an especially interesting case with regard to island phenomena. On the one hand, the various environmental differences just described, along with the possibility of transfer, could very reasonably be expected to lead to differences between bilinguals and monolinguals, especially with languages like English and Korean, where the island properties are so different. On the other hand, many have suggested that islands arise not because of learning *per se*, but because of resource limitations on the processor or computational limitations on the grammar, and if such is the case, we would expect few differences between monolinguals and bilinguals, assuming that their processing and/or grammatical resources are similar.

In this paper, we examine islands in heritage speakers of Korean in the United States, i.e., early Korean/English bilinguals for whom English has become the dominant language. If islands are susceptible to environmental influences, then there are many reasons to expect this type of bilingual to behave differently with regard to islands than monolingual speakers, as we have seen. If, on the other hand, islands are primarily the result of specific properties of the processor and/or grammar, then we would expect these bilinguals to display island behavior that is basically the same as monolinguals.

We will focus in particular on the heritage speakers' sensitivity to islands in Korean. As we have seen, Korean is a *wh in-situ* language and has been claimed to show island effects in *wh*-clauses, but not in adjunct clauses. English, on the other hand, has *wh*-movement and shows island effects in both *wh*-clauses and adjunct clauses. We perform the same set of formal acceptability experiments on native Korean speakers residing in Korea and on heritage speakers of Korean living in the U.S. As we will see, our experiments show that sensitivity to islands is very similar in the two groups, lending support to the idea that island phenomena are largely immune to environmental influences and stem from deeper properties of the processor and/or grammar.

The paper is structured as follows: Section Island Effects in Korean gives further details about the nature of island constraints in Korean, Section Experiments presents a series of four experiments probing this phenomenon in both native and heritage speakers, and Section Conclusion presents the overall conclusions.

## Island effects in korean

Given the lack of overt *wh*-movement in the language, the question of whether a given island effect obtains in Korean reduces to the question of whether it is possible for an *in-situ wh*-phrase within a putative island domain to take scope outside of that domain. For adjunct clauses, it is usually thought that such wide-scope readings are in fact possible, as in (5) above, and for this reason, adjunct clauses are often believed not to have island status in Korean. For *wh*-clauses, however, the facts are not as clear. Many have claimed that a wide-scope reading for a *wh*-phrase in such clauses, as in (3) above, is not possible (e.g., Lee, 1982; Kim, 1989; Nishigauchi, 1990; Han, 1992; Watanabe, 1992 for Japanese; Hong, 2004 for Korean), suggesting that these clauses are islands, but others have claimed that it is possible (e.g., Suh, 1987; Ishihara, 2002; Choi, 2006; Hwang, 2007 for Korean; Sprouse et al., 2011 for Japanese), suggesting that these clauses are not islands.

One of the reasons for this lack of clarity surrounding the status of *wh*-islands in the literature is the fact that simple acceptability judgments of the string are not sufficient to decide the matter. First, the issue is how the scope of the *wh*-phrase is interpreted, not whether the sentence is acceptable or not. (3) is uncontroversially acceptable, for instance, if one gives a narrow-scope reading to the *wh*-word, as in (6).

(6) Did Mary know who Obama met ___?

Second, in addition to their interpretation as interrogatives, bare *wh*-words in Korean may also be interpreted as existential pronouns. Thus, in addition to the two readings already given for (3), an interpretation as in (7) is also possible.

(7) Did Mary know whether Obama met someone?

Combined, these two facts mean that for any given question with a *wh*-word, if one reading is not available, another typically is, with the result that all such questions give the appearance of being acceptable. This has made exploring the possibility of island effects in Korean very difficult and has no doubt contributed to the lack of consensus in the literature regarding *wh*-islands in Korean. In this study, we are able to circumvent this problem by presenting participants with question-answer pairs and soliciting acceptability judgments on the answer, rather than the question. Given that the answer will be appropriate for one reading of the question but not others, we are thus able to obtain, albeit indirectly, an acceptability rating for a particular reading. Using this technique, we are able to accomplish two goals. First, we are able to establish clearly the extent to which island effects exist in Korean, despite the lack of clarity in the literature. Second, we are able to make precise comparisons in this regard between native speaker controls and heritage speakers.

## Experiments

The following four experiments use the technique just described to explore the possibility of island effects in Korean. We test both *wh*-clauses and adjunct clauses, using both native Korean speaker controls and heritage speakers of Korean (Korean/English bilinguals).

### Experiment 1: canonical *wh*-Islands in Korean

#### Participants

Twenty-eight English-dominant heritage speakers of Korean, all students at UCSD, participated for course credit. Thirty three percent of the heritage participants were US-born and 67% were Korean-born and moved to the U.S. from Korea before age 7 (*M:* 3 years old, *SD*: 2.7). Their mean age at the time of testing was 20 (range: 18–25, *SD*: 1.8). Fifty seven percent of the heritage speakers reported that Korean was their mother tongue, 33% reported English, and the remaining 10% reported both languages. 86% of the parents spoke only Korean with them, and 14% spoke both languages. All were literate in Korean. As a control group, 48 native speakers of Korean who were residing in Korea at the time of testing participated online (*M*: 28 years old, range: 20–34, *SD*: 3.7).

After the experiment, participants took a Korean proficiency test. The proficiency test consisted of a cloze test, and multiple choice questions on synonym-antonym. The proficiency test results indicated that heritage speakers (*M*: 78%, range: 50–100%, *SD*: 16.7) were significantly less proficient than native speakers (*M*: 96%, range: 88–100%, *SD*: 3.1) [*F*_(1, 74)_ = 59.1, *p* < 0.0001].

#### Stimuli

Since island effects in Korean can be tested only by examining speakers' interpretation of sentences (i.e., *wh*-scope), we will measure the felicity of Question-Answer pairs. Variants of this method have been used in several studies testing scope ambiguity of *wh-in-situ* (e.g., Pesetsky, 1987; Umeda, 2008; Kitagawa and Hirose, 2012). The specifics of the experimental design are as follows.

We present participants a set of a context, a question (containing an island configuration), and an answer. Then, instead of asking for the acceptability of the question, we ask them to rate the acceptability of the answer as a very first response to the *wh*-question. The answers consist of two types: either “*wh*-answers” or “yes/no answers,” “*Wh*-answers” are appropriate for a direct *wh*-question interpretation of the preceding question, while “yes/no answers” are appropriate for a yes/no question interpretation. The answers would thus encourage one reading or the other. The acceptability of *wh*-answers would reflect the possibility of the island-violating interpretation when a *wh*-word is interpreted as a *wh*-question word with scope outside the embedded clause. On the other hand, when the *wh*-word is interpreted as an indefinite pronoun, or as a true *wh*-word with scope over only the embedded clause (yielding an indirect question), a yes/no question results.

There were thus three factors (Location of *wh*-word, Structure of embedded clause, Answer type), with a total of eight conditions. Stimuli consisted of question-answer pairs, preceded by a context. All question sentences were biclausal. As we will see below, there is optionality in the position of embedded clauses in Korean, but in this experiment, all embedded clauses immediately precede the matrix verb. They differed as to the *Location* of the *wh*-word (matrix vs. embedded clause) and the *Structure* of the embedded clause [declarative (non-island) vs. interrogative (island)]. There were also two different types of answers, either “*wh*-answers” or “yes/no answers.” Sample stimuli are provided in (8)–(15). In (8)–(9), the *wh*-word is in the matrix clause and the embedded clause is declarative, while in (10)–(11), the embedded clause is interrogative. In (12)–(13), the *wh*-word is in an embedded clause that is declarative, while in (14)–(15), the embedded clause is interrogative.

(8) Q: **Nwukwu**-ka [Obama-ka Mary-ul   manna-ss-ta**-ko**]
who -Nom -Nom -Acc                   meet-Past-Decl-that
tul-ess-**ni**?
hear-Past-Q
“Who heard that Obama met Mary?” or
“Did somebody hear that Obama met Mary?”
A: *WH*-ANSWER: Hillary-ka “Hillary”

(9) Q: Same as (8).
A: YES-NO ANSWER: Ney, tul-ess-eyo “Yes, heard”

(10) Q: **Nwukwu**-ka [Obama-ka Mary-ul   manna-ss-nun-**ci**]
who -Nom -Nom -Acc                   meet-Past-Adn-Q
tul-ess-**ni**?
hear-Past-Q
“Who heard whether Obama met Mary?” or
“Did somebody hear whether Obama met Mary?”
A: *WH*-ANSWER: Hillary-ka “Hillary”

(11) Q: Same as (10).
A: YES-NO ANSWER: Ney, tul-ess-eyo “Yes, heard”

(12) Q: Mary-nun [Obama-ka **nwukwu**-ul   manna-ss-ta-**ko**]
-Top -Nom who -Acc                     meet-Past-Decl-that
tul-ess-**ni**?
hear-Past-Q
“Who did Mary hear that Obama met?” or
“Did Mary hear that Obama met somebody?”
A: *WH*-ANSWER: Hillary-lul “Hillary”

(13) Q: Same as (12).
A: YES-NO ANSWER: Ney, tul-ess-eyo “Yes, heard”

(14) Q: Mary-nun [Obama-ka **nwukwu**-ul   manna-ss-nun-**ci**]
-Top -Nom who -Acc                     meet-Past-Adn-Q
tul-ess-**ni**?
hear-Past-Q
“Who did Mary hear whether Obama met?” or
“Did Mary hear who Obama met?”
A: *WH*-ANSWER: Hillary-lul “Hillary”

(15) Q: Same as (14).
A: YES-NO ANSWER: Ney, tul-ess-eyo “Yes, heard”

All question-answer pairs were preceded by a context consisting of a situation (e.g., “at the White House”) and a list of people involved in the situation (e.g., “Mary, Obama, Hillary”). These contexts were designed to make the *wh*-answer pragmatically plausible, even when this interpretation of the question would violate an island. All experimental stimuli were in Korean, but the English translation was also provided for the context part for the heritage speakers.

Forty sets of experimental sentences were distributed using a Latin Square design among eight lists consisting of five tokens of each of the eight conditions. Each list included 63 fillers, for an experimental/filler ratio of 1:1.5. All fillers were questions, some with and some without a *wh*-pronoun, representing a wide range of acceptability. All lists were randomized.

In 30 of the 40 sets, the matrix verb was matched across all conditions in the set. In the remaining 10 sets, however, one verb is used with declarative complements and another verb with interrogative complements (e.g., *sayngkakhata* “think” with declaratives and *kungkumhata* “wonder” with interrogatives. This was due to the limited number of verbs (e.g., *tutta* “hear”) that can take both declarative and interrogative complements. The *wh*-word *nwukwu* “who” was used in all stimuli.

All stimuli were presented in written form and thus without an explicit indication of prosody. It appears that prosody is able to ameliorate some possible island effects in Japanese and Korean (e.g., Kitagawa, 2005), but it may not be able to eliminate them entirely (e.g., Hwang, 2007). In any event, since our goal here is to compare native speakers and heritage speakers, what matters is that the experimental stimuli be identical, and that condition is met.

#### Method

**Figure 1:**
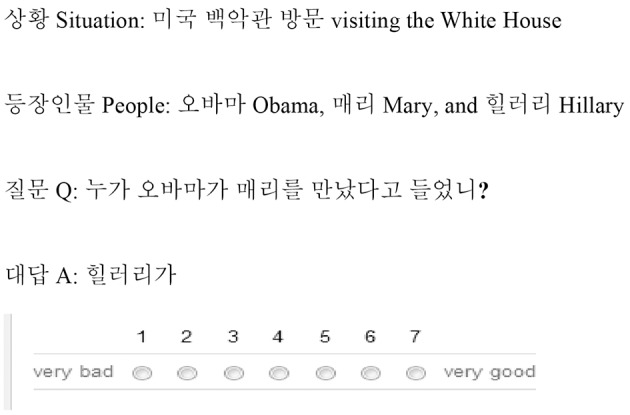
**An example of the experiment presentation in Experiment 1**.

The experiments were conducted in the Experimental Syntax Lab at UCSD for heritage speakers, and online for native speakers. The experiments in this study were approved by the Institutional Review Board of the University of California, San Diego (#110080). All subjects involved gave their informed written consent. Subjects were instructed to rate the acceptability of the answer as a first response to the question, using a 7-point scale (with 1 “very bad” and 7 “very good”). A sample item is given in Figure 1.

#### Analysis

Acceptability scores from each participant were z-score transformed prior to analysis, and a series of repeated-measures ANOVAs were conducted on the z-score results. Each group's data were separated by answer type, and separate repeated measures ANOVAs were run for each answer type in each group, with Location of *wh*-word (matrix vs. embedded) and Structure of embedded clause (non-island “declarative” vs. island “interrogative”) as within-subjects variables, and “subject” (F1) and “item” (F2) as random factors.

An interaction between Location and Structure, where the embedded *wh*-word in an interrogative clause is of lower acceptability than the other three conditions, will be suggestive of an island effect. In order to compare the effect size between groups of any such interaction, differences-in-differences scores (DD) are calculated as follows for each participant using the z-scores for the *wh*-answer type: DD = D1 (Non-Island/Embedded—Island/Embedded)—D2 (Non-Island/Matrix—Island/Matrix). A positive DD score signals super-additivity: the result is more than the sum of the two individual experimental factors. A larger DD score represents a larger island effect, while a negative DD score represents a sub-additive (non-island) interaction.

#### Results

**Figure 2:**
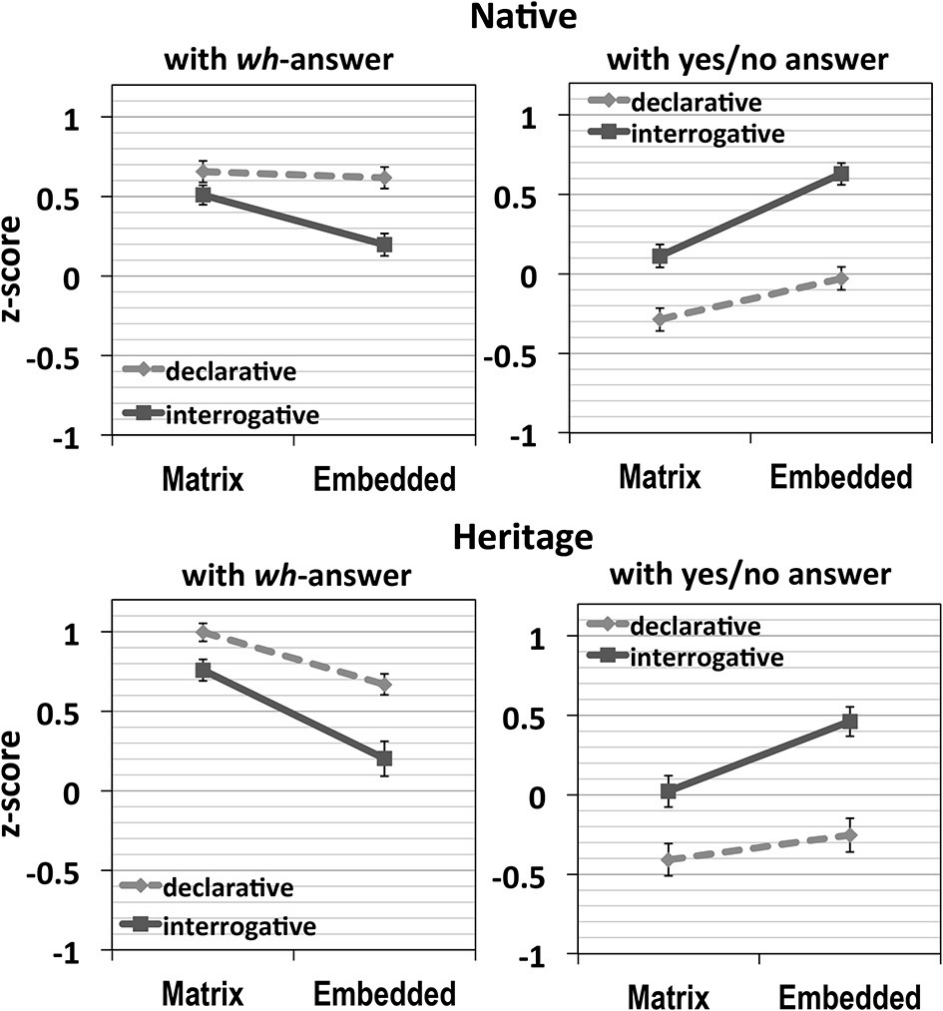
**Results of Experiment 1**.

The results are plotted in Figure 2 (error bars in all figures represent SE). The first two graphs are natives' results and the following two graphs are heritage speakers'. In both groups, the left graph represents the acceptability of *wh*-answers and the right graph shows that of yes/no answers.

First, with *wh*-answers, in the results of both groups, when a *wh*-word is located in the matrix clause, the two types of structures were rated similarly, but with an embedded *wh*-word, the declarative condition was preferred over the interrogative condition, indicating dispreference for the matrix *wh*-scope of the embedded *wh*-word, that is the *wh*-island effect. This was also shown by significant main effects for Location [native: *F1*_(1, 47)_ = 10.17, *p* = 0.003, *F2*_(1, 39)_ = 15.22, *p* < 0.0001; heritage: *F1*_(1, 27)_ = 27.66, *p* < 0.0001, *F2*_(1, 39)_ = 44.14, *p* < 0.0001], and Structure [native: *F1*_(1, 47)_ = 29.83, *p* < 0.0001, *F2*_(1, 39)_ = 28.12, *p* < 0.0001; heritage: *F1*_(1, 27)_ = 48.86, *p* < 0.0001, *F2*_(1, 39)_ = 32.57, *p* < 0.0001]. The interaction between these two factors was significant for natives [*F1*_(1, 47)_ = 16.17, *p* < 0.0001, *F2*_(1, 39)_ = 7.13, *p* = 0.011], and marginal for heritage speakers [*F1*_(1, 27)_ = 3.47, *p* = 0.07, *F2*_(1, 39)_ = 3.41, *p* = 0.07].

The differences-in-differences (DD) scores in both groups were positive [Native: 0.28 (*SD*: 0.48), Heritage: 0.23 (*SD*: 0.65)], indicating a super-additive *wh*-island effect in both groups. A one-way ANOVA with DD-score as a dependent factor, and Group as a fixed factor yielded no significant difference between the two groups (*p* = 0.71).

With yes/no answers, the pattern was reversed, with higher acceptability with embedded *wh*-words, than with matrix *wh*-words. Crucially, the condition with an embedded *wh*-word inside a *wh*-clause was preferred to be answered with yes/no answers, more than in any other conditions, indicating a wh- island effect. Both groups displayed main effects for Location [native: *F1*_(1, 47)_ = 33.64, *p* < 0.0001, *F2*_(1, 39)_ = 39.21, *p* < 0.0001; heritage: *F1*_(1, 27)_ = 18.08, *p* < 0.0001, *F2*_(1, 39)_ = 19.74, *p* < 0.0001], and Structure [native: *F1*_(1, 47)_ = 76.08, *p* < 0.0001, *F2*_(1, 39)_ = 61.83, *p* < 0.0001; heritage: *F1*_(1, 27)_ = 54.66, *p* < 0.0001, *F2*_(1, 39)_ = 71.96, *p* < 0.0001]. In addition, for natives, the interaction of Location and Structure was significant in the subjects analysis and close to significant in the items analysis [*F1*_(1, 47)_ = 5.04, *p* = 0.03, *F2*_(1, 39)_ = 3.89, *p* = 0.056], while for heritage speakers, the interaction approached significance in both types of analysis [*F1*_(1, 27)_ = 3.89, *p* = 0.059, *F2*_(1, 39)_ = 3.15, *p* = 0.08].

In sum, these results suggest a very clear *wh*-island effect in Korean for the natives. That is, when the *wh*-word is located within an embedded interrogative clause, the *wh*-answer is strongly dispreferred and a yes/no answer is strongly preferred. Since the *wh*-answer is only compatible with matrix scope for the *wh*-word and the yes/no answer is only compatible with embedded scope, these results suggest that the *wh*-word is not able to scope out of the embedded interrogative clause. For heritage speakers, the situation is less clear. They exhibit a numerically similar pattern suggestive of a *wh*-island effect, but this effect does not reach significance. We return to this issue in Experiment 3, where we test for the existence of a *wh*-island effect in the two populations by means of stimuli where the interrogative clause is in sentence-initial position, as is also possible in Korean.

### Experiment 2: acceptability of canonical adjunct-islands in korean

#### Participants, method, and analysis

The participants, method, and analysis of the results were the same as in Experiment 1.

#### Stimuli

The basic design of the experiment is the same as in Experiment 1, consisting of a total of 8 conditions, reflecting three factors: Location of *wh*-word (matrix vs. embedded) × Structure of embedded clause [complement (non-island) vs. adjunct (island)] × Answer type (*wh*-answer vs. yes/no-answer). What distinguishes this experiment from the previous one is that here we are contrasting embedded complement clauses with embedded adjunct clauses. As in Experiment 1, all embedded clauses immediately precede the verb here, although other positions are also possible (see Experiments 3 and 4 below).

All 8 conditions in this experiment were lexically matched except for the matrix verb, which had to differ between complement clauses and adjunct clauses for selectional reasons (e.g., *tutta* “hear” in complement conditions vs. *natanata* “appear” in adjunct conditions).

As in Experiment 1, 40 sets of experimental sentences were distributed using a Latin Square design among eight lists consisting of five tokens of each of the eight conditions. Each list included 63 fillers, for an experimental/filler ratio of 1:1.5. All lists were randomized. The *wh*-word *nwukwu* “who” was used in all stimuli. Sample stimuli are provided in (16)–(23).

(16) Q: **Nwukwu**-ka [Obama-ka Mary-ul   manna-ss-ta**-ko**]
who -Nom -Nom -Acc                   meet-Past-Decl-that
tul-ess-**ni**?
hear-Past-Q
“Who heard that Obama met Mary?” or
“Did somebody hear that Obama met Mary?”
A: *WH*-ANSWER: Hillary-ka “Hillary”

(17) Q: Same as (16).
A: YES-NO ANSWER: Ney, tul-ess-eyo “Yes, heard”

(18) Q: **Nwukwu**-ka [Obama-ka Mary-ul   manna-ss-ul-**ttay**]
who -Nom -Nom -Acc                   meet -Past-Adn-when
natana-ss-**ni**?
appear-Past-Q
“Who appeared when Obama met Mary?” or
“Did somebody appear when Obama met Mary?”
A: *WH*-ANSWER: Hillary-ka “Hillary”

(19) Q: Same as (18).
A: YES-NO ANSWER: Ney, natana-ss-eyo “Yes, appeared”

(20) Q: Mary-nun [Obama-ka **nwukwu**-ul   manna-ss-ta-**ko**]
-Top -Nom who -Acc                     meet-Past-Decl-that
tul-ess-**ni**?
hear-Past-Q
“Who did Mary hear that Obama met?” or
“Did Mary hear that Obama met somebody?”
A: *WH*-ANSWER: Hillary-lul “Hillary”

(21) Q: Same as (20).
A: YES-NO ANSWER: Ney, tul-ess-eyo “Yes, heard”

(22) Q: Mary-nun [Obama-ka **nwukwu**-ul   manna-ss-ul-**ttay**]
-Top -Nom who -Acc                     meet-Past-Adn-when
natana-ss-**ni**?
appear-Past-Q
“Who did Mary appear when Obama met?” or
“Did Mary appear when Obama met somebody?”
A: *WH*-ANSWER: Hillary-lul “Hillary”

(23) Q: Same as (22).
A: YES-NO ANSWER: Ney, natana-ss-eyo “Yes, appeared”

#### Results

**Figure 3:**
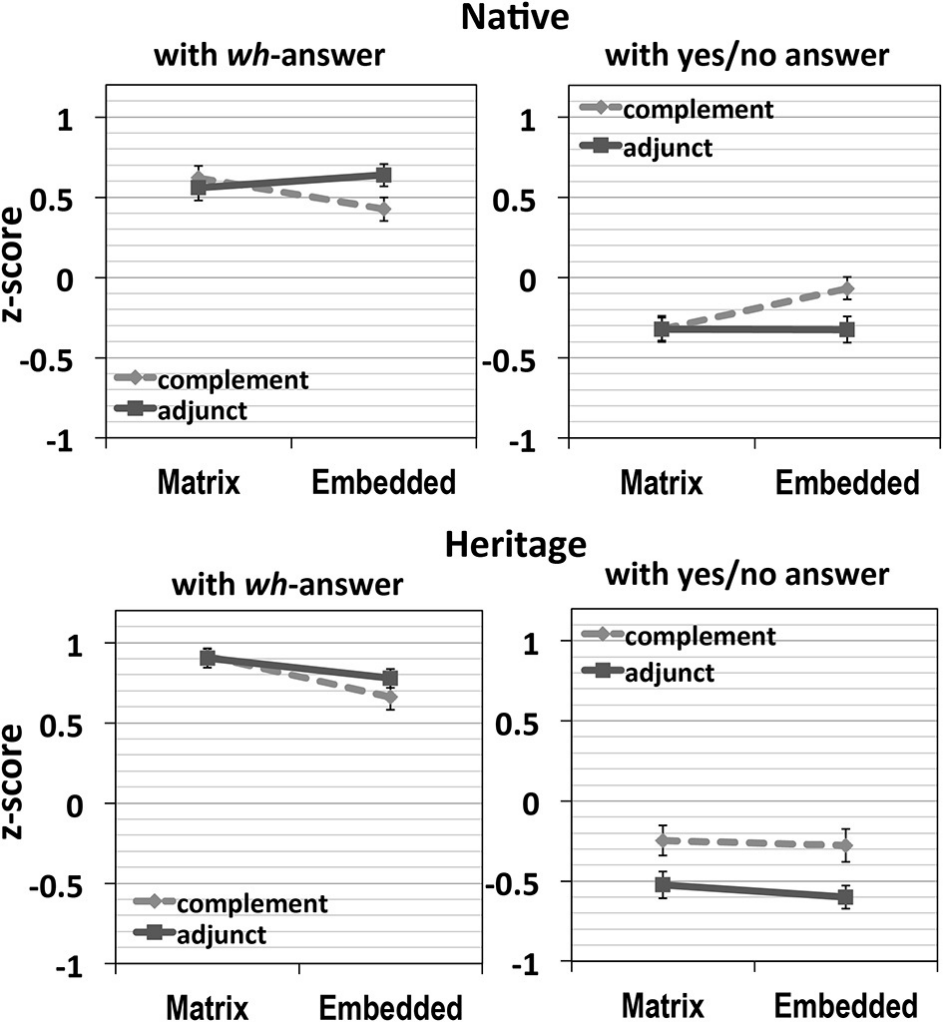
**Results of Experiment 2**.

In Figure 3, the first two graphs represent natives', and the following two graphs are heritage speakers' results. In each set of graphs, the first graph shows the results with the *wh*-answer, and the second graph displays the results with the yes/no answer.

The acceptability of the adjunct clause conditions did not change much depending on the location of the *wh*-word with both types of answers in both groups, indicating the absence of adjunct island effects. First, for the heritage speakers, a *wh*-word within an adjunct clause does not result in significantly decreased acceptability with *wh*-answers or increased acceptability with yes/no answers, as may be seen in the lack of an interaction between Structure and Location [with *wh*-answer: *F1*_(1, 27)_ = 1.49, *p* = 0.23, *F2*_(1, 39)_ = 1.61, *p* = 0.21; with yes/no answer: *F1*_(1, 27)_ = 0.14, *p* = 0.71, *F2*_(1, 39)_ = 0.19, *p* = 0.66].

The results are similar for the native speakers in that there is no evidence of any adjunct island effect. However, the native group showed a main effect of Structure on the yes/no answers [*F1*_(1, 47)_ = 8.52, *p* = 0.005, *F2*_(1, 39)_ = 8.25, *p* = 0.007], as well as a mostly significant interaction of Structure and Location with both types of answers [with *wh*- answer: *F1*_(1, 47)_ = 12.05, *p* = 0.001, *F2*_(1, 39)_ = 4.54, *p* = 0.039; with yes/no answer: *F1*_(1, 47)_ = 6.31, *p* = 0.016, *F2*_(1, 39)_ = 3.04, *p* = 0.089]. Nevertheless, the direction of the interaction was the opposite of what one would expect for a classic island effect: the condition in which the *wh*-word is located within an adjunct clause was rated the highest out of the four conditions with *wh*-answers, and the lowest with yes/no answers. There is thus no sign of an adjunct island effect for this group.

The differences-in-differences (DD) scores with *wh*-answer were also negative in both groups [native controls: −0.28 (*SD*: 0.56), heritage speakers: −0.13 (*SD*: 0.57)], with no significant difference between the groups. This confirms again no super-additive adjunct island effects in Korean for both groups.

In sum, the reverse interaction of Location and Structure in the native group and the absence of interaction in the heritage group thus very strongly suggest that there are no adjunct island effects in Korean for either group of speakers.

### Interim summary

In Experiments 1 and 2 with canonically ordered embedded interrogative and adjunct clauses, we found *wh*-island effects, but no adjunct island effects in Korean. The *wh*-island violating condition in Experiment 1 was the least acceptable compared to other conditions, while the adjunct island violating condition was rated similarly with its counterparts. The results of the native and heritage groups were similar, thus suggesting that the development of (non-)island effects is largely independent of the learning environment.

In Experiments 3 and 4, we will attempt to replicate these results with different groups of participants and different types of stimuli. The embedded clauses in these experiments will be scrambled to a sentence-initial position. Since this is a natural position for embedded clauses in Korean, and the preferred position for adjunct clauses, it is possible that this will allow for a fairer test for the presence of island effects.

### Experiment 3: acceptability of scrambled *wh*-islands in korean

#### Participants

Nineteen English-dominant heritage speakers of Korean, all students at UCSD, participated for course credit. 27% of the heritage participants were US-born and 73% were Korean-born and moved to the U.S. from Korea before age 7 (*M*: 3 years old, *SD*: 2.7). Their mean age at the time of testing was 20 (range: 19–23, *SD*: 1.2). 53% of the heritage speakers reported that Korean was their mother tongue, 21% reported English, and the remaining 26% reported both languages. 85% of the parents spoke only Korean with them, and 15% spoke both languages. 48 native speakers of Korean residing in Korea served as a control group (*M*: 26 years old, range: 20–37, *SD*: 4.8).

After the experiment, participants took the Korean proficiency test, the same one used in Experiments 1 and 2. The proficiency test results implied that heritage speakers (*M*: 78%, range: 51–94%, *SD*: 13.6) were significantly less proficient than native speakers (*M*: 96%, range: 88–100%, *SD*: 3.6) [*F*_(1, 65)_ = 76.2, *p* < 0.0001].

#### Stimuli, method, and analysis

The stimuli differed from those in Experiment 1 only by the location of the embedded clauses: the embedded clauses in this experiment were sentence-initial, whereas those in Experiment 1 were in their canonical (center-embedded) position. There were 8 experimental conditions reflecting 3 factors, just as in Experiment 1: Location of *wh*-word (matrix clause vs. embedded clause) × Structure of embedded clause (declarative vs. interrogative) × Answer type (*wh*-answer vs. yes/no-answer). Sample stimuli are provided in (24)–(31). The methods and analysis of the results were the same as in Experiment 1.

(24) Q: [Obama-ka Mary-ul   manna-ss-ta**-ko**]         **nwukwu**-ka
-Nom -Acc                meet-Past-Decl-that    who -Nom
tul-ess-**ni**?
hear-Past-Q
“Who heard that Obama met Mary?” or
“Did somebody hear that Obama met Mary?”
A: *WH*-ANSWER: Hillary-ka “Hillary”

(25) Q: Same as (24).
A: YES-NO ANSWER: Ney, tul-ess-eyo “Yes, heard”

(26) Q: [Obama-ka Mary-ul    manna-ss-nun-**ci**]    **nwukwu**-ka
-Nom -Acc                 meet-Past-Adn-Q   who -Nom
tul-ess-**ni**?
hear-Past-Q
“Who heard whether Obama met Mary?” or
“Did somebody hear whether Obama met Mary?”
A: *WH*-ANSWER: Hillary-ka “Hillary”

(27) Q: Same as in (26).
A: YES-NO ANSWER: Ney, tul-ess-eyo “Yes, heard.”

(28) Q: [Obama-ka **nwukwu**-ul   manna-ss-ta-**ko**]          Mary-ka
-Nom who -Acc              meet-Past-Decl-that    -Nom
tul-ess-**ni**?
hear-Past-Q
“Who did Mary hear that Obama met?” or
“Did Mary hear that Obama met somebody?”
A: *WH*-ANSWER: Hillary-lul “Hillary”

(29) Q: Same as (28).
A: YES-NO ANSWER: Ney, tul-ess-eyo “Yes, heard”

(30) Q: [Obama-ka **nwukwu**-ul   manna-ss-nun-**ci**]     Mary-ka
-Nom who -Acc              meet-Past-Adn-Q   -Nom
tul-ess-**ni**?
hear-Past-Q
“Who did Mary hear whether Obama met?” or
“Did Mary hear who Obama met?”
A: *WH*-ANSWER: Hillary-lul “Hillary”

(31) Q: Same as in (30).
A: YES-NO ANSWER: Ney, tul-ess-eyo “Yes, heard.”

#### Results

**Figure 4:**
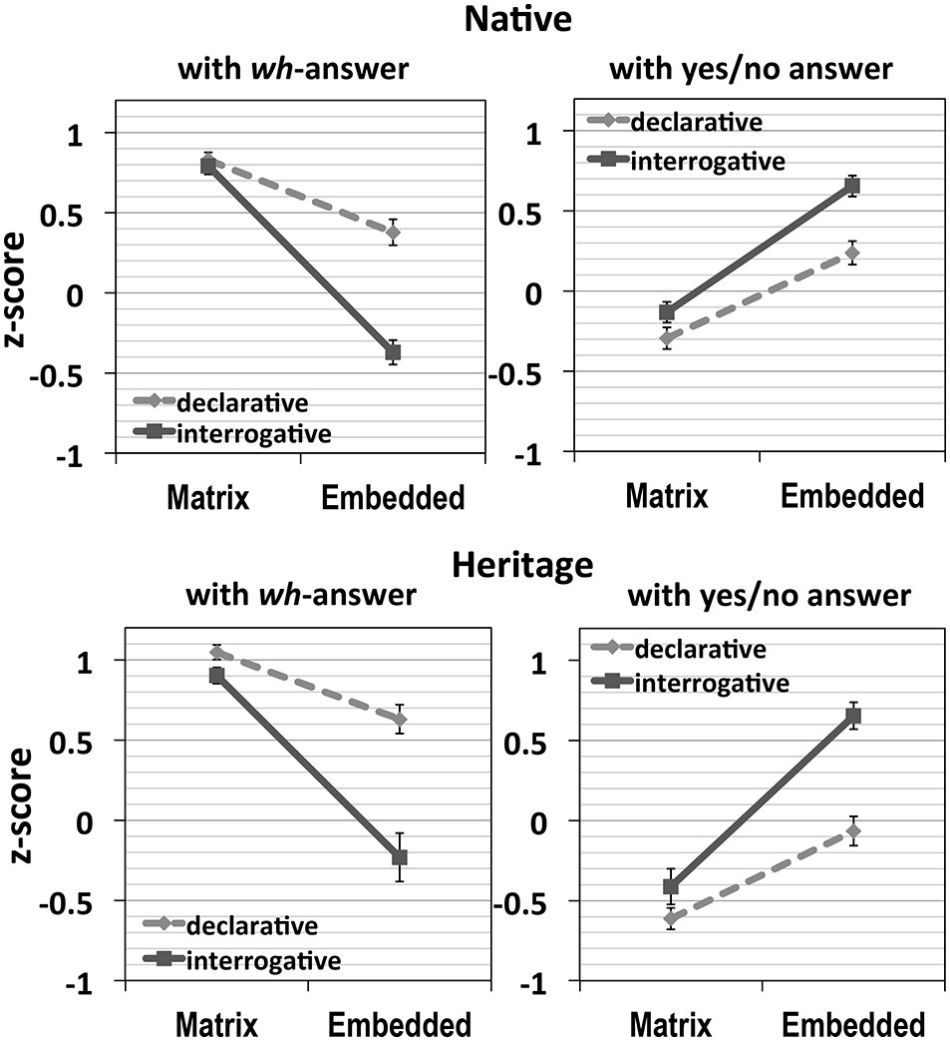
**Results of Experiment 3**.

Similar to the results in Experiment 1 on the *wh*-island effect with a canonically ordered interrogative clause, results in Experiment 3, presented in Figure 4, showed the *wh*-island effect with a sentence-initial interrogative clause in both native and heritage groups, but the effect was more robust in Experiment 3. In the results with *wh*-answer, there was no effect of the complement clause type when the *wh*-word is located in the matrix clause, in that all questions with a matrix *wh*-word were rated similarly regardless of the types of embedded clauses.

On the other hand, with an embedded *wh*-word, the island condition was significantly less preferred than the declarative condition. Also, the questions with an interrogative clause showed a distinctive acceptability depending on the location of the *wh*-word, that is the island violating condition was much less acceptable than its counterpart. This all suggests the *wh*-island effect in Korean, which is also supported by the statistical results as in the following.

First, natives exhibited main effects of Location [with *wh*-answers *F1*_(1, 47)_ = 183.01, *p* < 0.0001, *F2*_(1, 39)_ = 260.41, *p* < 0.0001]; with yes/no answers [*F1*_(1, 47)_ = 85.11, *p* < 0.0001, *F2*_(1, 39)_ = 167.63, *p* < 0.0001], and Structure [with *wh*-answers *F1*_(1, 47)_ = 48.57, *p* < 0.0001, *F2*_(1, 39)_ = 63.24, *p* < 0.0001]; with yes/no answers [*F1*_(1, 47)_ = 28.67, *p* < 0.0001, *F2*_(1, 39)_ = 29.80, *p* < 0.0001], as well as a significant interaction of Location and Structure [with *wh*-answers, *F1*_(1, 47)_ = 42.46, *p* < 0.0001, *F2*_(1, 39)_ = 42.15, *p* < 0.0001; with yes/no answers, *F1*_(1, 47)_ = 6.12, *p* = 0.017, *F2*_(1, 39)_ = 5.86, *p* = 0.02].

Heritage speakers displayed very similar results, showing main effects of Location [with *wh*-answers *F1*_(1, 18)_ = 59.53, *p* < 0.0001, *F2*_(1, 39)_ = 68.70, *p* < 0.0001]; with yes/no answers [*F1*_(1, 18)_ = 87.09, *p* < 0.0001, *F2*_(1, 39)_ = 67.79, *p* < 0.0001], and Structure [with *wh*-answers *F1*_(1, 18)_ = 48.64, *p* < 0.0001, *F2*_(1, 39)_ = 47.29, *p* < 0.0001]; with yes/no answers [*F1*_(1, 18)_ = 101.65, *p* < 0.0001, *F2*_(1, 39)_ = 34.28, *p* < 0.0001], as well as a significant interaction of Location and Structure [with *wh*-answers, *F1*_(1, 18)_ = 26.33, *p* < 0.0001, *F2*_(1, 39)_ = 42.15, *p* < 0.0001; with yes/no answers, *F1*_(1, 18)_ = 17.30, *p* = 0.002, *F2*_(1, 39)_ = 12.45, *p* = 0.001].

The two groups' island effect size with *wh*-answers, indicated by the differences-in-differences (DD) scores, were very similar to each other [native: 0.71 (SD: 0.75), heritage: 0.72 (SD: 0.61)].

The significant interaction between Location and Structure suggests a strong *wh*-island effect in Korean for both groups. When the *wh*-word is within an embedded interrogative clause, acceptability drops for the *wh*-answer and rises for the yes/no answer, as we would expect if the *wh*-word is unable to take scope out of that clause.

### Experiment 4: acceptability of scrambled adjunct-islands in korean

#### Participants

The participants in this experiment were the same as in Experiment 3.

#### Stimuli, method, and analysis

The stimuli in this experiment were the same as those in Experiment 2, but with sentence-initial embedded clauses. There was a total of 3 factors with 8 conditions: Location of *wh*-word (matrix clause vs. embedded clause) × Structure of embedded clause (complement vs. adjunct) × Answer type (*wh*-answer vs. yes/no-answer). Sample stimuli are presented in (32)–(39). The method and analysis were identical to Experiment 2.

(32) Q: [Obama-ka Mary-ul   manna-ss-ta**-ko**]               **nwukwu**-ka
-Nom -Acc                meet-Past-Decl-that          who -Nom
tul-ess-**ni**?
hear-Past-Q
“Who heard that Obama met Mary?” or
“Did somebody hear that Obama met Mary?”
A: *WH*-ANSWER: Hillary-ka “Hillary”

(33) Q: Same as (32).
A: YES-NO ANSWER: Ney, tul-ess-eyo “Yes, heard”

(34) Q: [Obama-ka Mary-ul     manna-ss-ul-**ttay**]        **nwukwu**-ka
-Nom -Acc                  meet-Past-Adn-when   who -Nom
natana-ss-**ni**?
appear-Past-Q
“Who appeared when Obama met Mary?” or
“Did somebody appear when Obama met Mary?”
A: *WH*-ANSWER: Hillary-ka “Hillary”

(35) Q: Same as in (34).
A: YES-NO ANSWER: Ney, natana-ss-eyo “Yes, appeared”

(36) Q: [Obama-ka **nwukwu**-ul   manna-ss-ta-**ko**]          Mary-ka
-Nom who -Acc              meet-Past-Decl-that     -Nom
tul-ess-**ni**?
hear-Past-Q
“Who did Mary hear that Obama met?” or
“Did Mary hear that Obama met somebody?”
A: *WH*-ANSWER: Hillary-lul “Hillary”

(37) Q: Same as (36).
A: YES-NO ANSWER: Ney, tul-ess-eyo “Yes, heard”

(38) Q: [Obama-ka **nwukwu**-ul    manna-ss-ul-**ttay**]        Mary-ka
Nom who -Acc                meet-Past-Adn-when    -Nom
natana-ss-**ni**?
appear-Past-Q
“Who did Mary appear when Obama met?” or
“Did Mary appear when Obama met somebody?”
A: *WH*-ANSWER: Hillary-lul “Hillary”

(39) Q: Same as in (38).
A: YES-NO ANSWER: Ney, natana-ss-eyo “Yes, appeared”

#### Results

**Figure 5:**
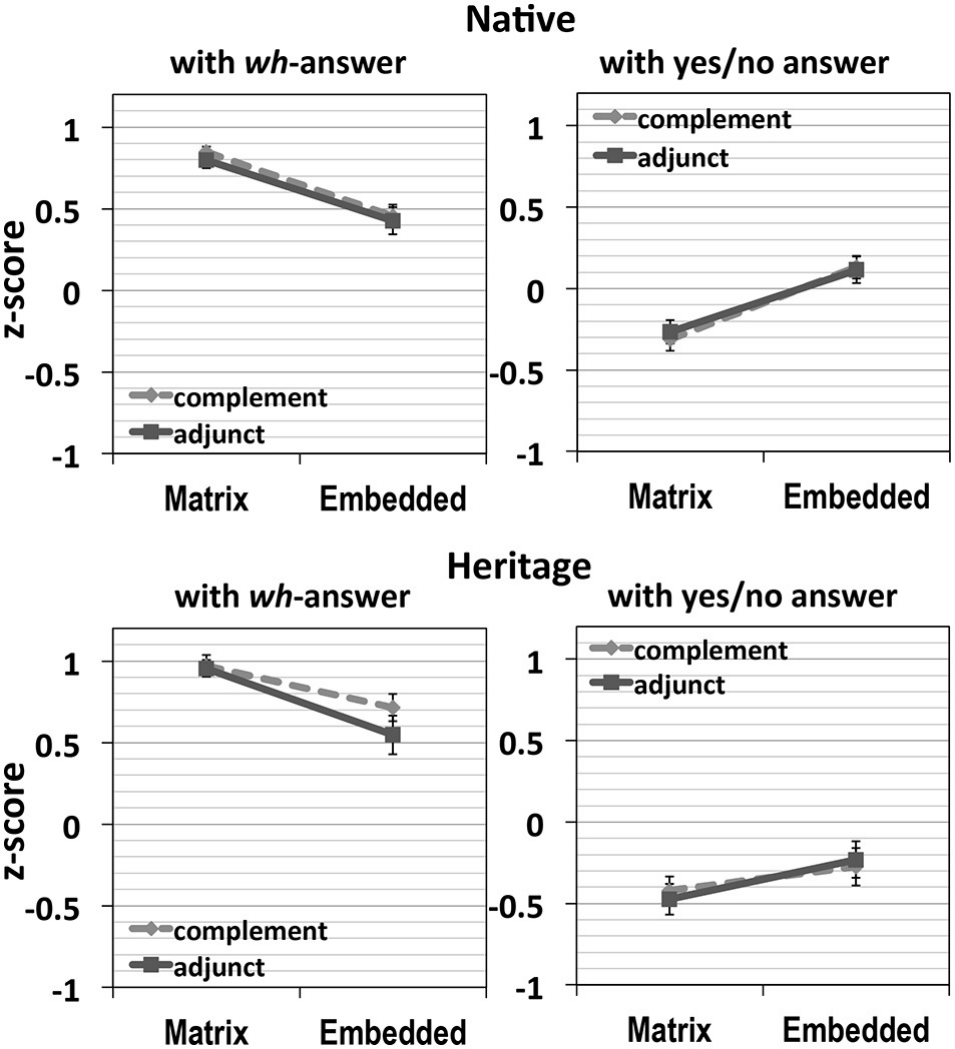
**Results of Experiment 4**.

As plotted in Figure 5, no adjunct island effect was found in either group. Both complement and adjunct clauses received similar acceptability. First, native speakers showed a significant main effect of Location with both *wh*-answers [*F1*_(1, 47)_ = 35.02, *p* < 0.0001, *F2*_(1, 39)_ = 40.09, *p* < 0.0001] and yes/no answers [*F1*_(1, 47)_ = 39.79, *p* < 0.0001, *F2*_(1, 39)_ = 47.91, *p* < 0.0001]. Heritage speakers also revealed a main effect of Location, but the effect was significant only with *wh*-answers [*F1*_(1, 18)_ = 10.28, *p* = 0.005, *F2*_(1, 39)_ = 27.99, *p* < 0.0001] and marginal with yes/no answers [*F1*_(1, 18)_ = 3.26, *p* = 0.088, *F2*_(1, 39)_ = 3.99, *p* = 0.053]. Crucially, neither a main effect of Structure nor an interaction between Location and Structure was significant with either answer type for either group. The differences-in-differences (DD) scores with *wh*-answers were very close to zero in both groups [native: −0.06 (*SD*: 0.78), heritage: −0.09 (*SD*: 0.57)].

The results here provide further support for the conclusion reached in Experiment 2 that there are no adjunct island effects in Korean for either group. The lack of an interaction between Location and Structure suggests that there is no restriction on *wh*-words in adjunct clauses taking wide scope, i.e., that there is no adjunct island.

### Summary of the results in experiments 1–4

Statistical results of *wh*-answers in Experiments 1–4 are summarized in Table 1. As mentioned in Section Stimuli, the results of *wh*-answers reflect the acceptability of the direct *wh*-question reading where all the *wh*-words are interpreted as *wh*-question words. On the other hand, the results of yes/no answers, specifically with *that*-clauses, indicate the preferred reading of a *wh*-word, either as a question word or as an existential pronoun (i.e., someone) with a *that*-complement clause, while with an interrogative clause, yes/no answers are when the *wh*-word is interpreted either as an indefinite pronoun, or as a true *wh*-word with scope over only the embedded clause (yielding an indirect question). For this reason, direct comparison of the acceptability of yes/no answers between a declarative clause and an interrogative clause may not be very meaningful with regard to the issue of island effects in Korean. Thus, the evaluation of island effects in Korean will be primarily based on the results of the *wh*-answers here.

**Table 1:** **Results summary of *Wh*-answer in Experiments 1–4**.

**Group**		**Native controls**	**Heritage speakers**
*N*		48	28
Experiment 1 Canonically ordered Interrogative clause	Structure	✔ / ✔	✔ / ✔
	Location	✔ / ✔	✔ / ✔
	Interaction	✔ / ✔	#/#
	z-score (island condition)	0.19	0.22
	Raw score (island condition)	4.36 (1.48)	4.52 (1.31)
	DD score	0.28	0.23
Experiment 2 Canonically ordered Adjunct clause	Structure	✘ / ✘	✘ / ✘
	Location	✘ / ✘	✘ / ✘
	Interaction	✔ / ✔	✘ / ✘
	z-score (island condition)	0.66	0.77
	Raw score (island condition)	5.43 (1.44)	6.02 (0.89)
	DD score	−0.28	−0.13
*N*		48	19
Experiment 3 Scrambled Interrogative clause	Structure	✔ / ✔	✔ / ✔
	Location	✔ / ✔	✔ / ✔
	Interaction	✔ / ✔	✔ / ✔
	z-score (island condition)	−0.37	−0.06
	Raw score (island condition)	2.95 (1.38)	3.21 (1.65)
	DD score	0.71	0.72
Experiment 4 Scrambled Adjunct clause	Structure	✘ / ✘	✘ / ✘
	Location	✔ / ✔	✔ / ✔
	Interaction	✘ / ✘	✘ / ✘
	z-score (island condition)	0.42	0.55
	Raw score (island condition)	4.78 (1.55)	5.24 (1.45)
	DD score	−0.06	−0.09

✔ means “significant” (p < 0.05), # means “marginal” (p < 0.1), ✘ means “insignificant” (p > 0.1), by-subject analysis on the left, by-item analysis on the right.

Overall, the results of native and heritage speakers were similar in that both groups showed *wh*-island effects in Experiments 1 and 3, but no adjunct island effects in Experiments 2 and 4. In both Experiments 1 and 3, the condition in which the *wh*-word was within the embedded *wh*-clause was noticeably worse than other conditions, indicating *wh*-island effects, which was shown by a significant interaction between the two factors, Location of *wh*-word (matrix or embedded clause) and Structure of embedded clause type (non-island or island). For heritage speakers, the effect was only marginal in Experiment 1, but it reached significance in Experiment 3. For native speakers also, the effect was smaller in Experiment 1 (DD = 0.28) than in Experiment 3 (DD = 0.71), though it was significant in both experiments. On the other hand, in Experiments 2 and 4, the acceptablity of the island-violating condition (i.e., Embedded *wh*-word inside the adjunct clause) was similar to its counterpart with the embedded *that*-clause, and no significant island effect was found. Native speakers in Experiment 2 did show an interaction between Location and Structure, but in the opposite direction of what would be expected for an island effect.

## Conclusion

Two very clear conclusions emerge from the results that we have seen in the experiments just presented. First, *wh*-clauses and adjunct clauses appear to behave very differently in Korean: *wh*-clauses behave like islands (i.e., *wh*-words within them may not take scope outside of that clause), while adjunct clauses do not (i.e., *wh*-words within them are easily able to take scope outside of that clause). This result is important in itself, because as we saw in Section Island Effects in Korean, there has been considerable uncertainty in the literature about the status of *wh*-islands in Korean.

Second, heritage speakers of Korean show essentially the same island behavior as the native controls. This was especially true in Experiments 3 and 4, where the embedded clause was scrambled and the results between the two groups were virtually identical, but even in Experiments 1 and 2, where the embedded clause was not scrambled, the two groups' results are very similar. Both heritage and native speakers thus appear to treat *wh*-clauses as islands and adjunct clauses as non-islands.

This second conclusion is particularly striking for a number of reasons. As we saw earlier, the learning environment for native and heritage speakers can be very different and this often leads to very clear language differences. Heritage speakers presumably have less overall exposure to Korean, and what exposure they have may be more limited in scope (e.g., coming from only a few speakers, rather than an entire community). In addition, heritage speakers' acquisition of Korean may have been incomplete and they may also have undergone attrition in the years since childhood. Beyond these factors relating to Korean itself, heritage speakers are also likely to be susceptible to transfer effects from English, their dominant language. It is relevant to note here that the island facts of English are different (*wh*-clauses and adjunct clauses are both islands in English), and in separate work, we have shown that these heritage speakers have native-like sensitivity to islands in English as well (Kim, 2015).

For all of these reasons, one would very reasonably expect that native and heritage speakers would differ with regard to island behavior in Korean, as they do for many other types of linguistic phenomena, but as we have seen, this is not the case. This result is consistent with the view that island phenomena are not learned, but rather follow from constraints on the way that the processor and/or grammar operates. What specific constraints could result in the type of island phenomena that we observe here for Korean? We do not offer a definitive solution here, but we do note that some very plausible possibilities have been proposed in the literature. In terms of processing, for instance, it has been claimed that a *wh*-word and a question marker need to form a dependency in *wh-in-situ* languages that is similar to the more familiar filler-gap dependency in *wh*-movement languages, and that this dependency needs to be completed as soon as possible (e.g., Miyamoto and Takahashi, 2002; Aoshima et al., 2003; Ueno and Kluender, 2009; Sprouse et al., 2011). If this dependency determines the scope of the *wh*-word, it then follows that in *wh*-clauses, scope will always be limited to that clause, since the search for a question marker will always be satisfied within that clause. This would result in the *wh*-island effect seen in Experiments 1 and 3. In adjunct clauses, on the other hand, there is no such question marker and the search continues until it is resolved outside of the adjunct clause. This leads to the lack of an island effect with adjunct clauses, as seen in Experiments 2 and 4. Alternatively, it could be that this dependency between the *wh*-word and the question marker is determined by the grammar and constrained by locality restrictions on it, as in Shimoyama's (2006) proposal for Japanese, in which the *wh*-word must associate with the question marker that is structurally closer. In this case too, though, the asymmetry in island behavior between *wh*-clauses and adjunct clauses results from the presence of a question marker in the former, but not in the latter.

Specifics aside, both of these approaches suggest that the (non-)island status of *wh*-clauses and adjunct clauses in Korean follows from fundamental properties of how the processor or the grammar operates. That is, *wh*-island violations are not possible in Korean because doing this would require a processing/grammatical operation beyond the capabilities of speakers. If this is correct, then the similarities that we have seen here between native and heritage speakers of Korean are not surprising. If heritage speakers were to not show native-like *wh*-island effects, this would suggest that they are somehow able to surpass the processing and/or grammatical capabilities of native speakers, which hardly seems plausible. With adjunct clauses, in contrast, nothing prevents either the native or the heritage speakers from computing wide-scope readings for the *wh*-word, so neither group shows adjunct island effects. The results that we have obtained are thus exactly what is predicted by approaches in which island behavior is simply the consequence of deeper processing/grammatical traits.

If, on the other hand, island phenomena did not follow from fundamental properties of the processor and/or grammar but instead were learned from the environment, we would not predict that native and heritage island behavior would necessarily be the same. They could be, of course, but given the many differences discussed earlier in the learning environment and the possibility of transfer, it seems likely that some differences in island behavior would emerge. Since this is not what was found, our results do not lend support to this approach.

## Author contributions

BK performed the detailed design and implementation of the experiments. BK and GG both participated in the overall conception, analysis, and interpretation of the experiments, and in the drafting and revision of this article.

### Conflict of interest statement

The authors declare that the research was conducted in the absence of any commercial or financial relationships that could be construed as a potential conflict of interest.
