# Supplementary material for: Islands and Non-islands in Native and Heritage Korean
Source: Front Psychol. 2016 Feb 15;7:134. doi: 10.3389/fpsyg.2016.00134 (PMC4753330; doi:10.3389/fpsyg.2016.00134)
Supplement: Supplementary file 1 [file DataSheet1.PDF]

Appendix: Sample Stimuli

Experiment 1: cannonical *wh*-island

Condition 1: a matrix *wh*-word with a *that*-clause
누가 수지가 반장을 좋아했다고 말했니?
누가 피터팬이 팅커벨을 응원했다고 전했니?
누가 강아지가 준호를 쫓아갔다고 들었니?
누가 크리스가 기자를 때렸다고 기억했습니까?
누가 고아들이 장애인들을 보살폈다고 알게 되었니?
누가 미애가 선생님을 속였다고 의심했습니까?
누가 지니가 감독을 죽였다고 밝혔습니까?
누가 대통령이 비서를 사랑했다고 눈치챘습니까?
누가 유엔이 간호사를 파견했다고 발표했습니까?
누가 오빠가 동생을 꼬집었다고 알아냈니?
누가 할머니가 할아버지를 걱정했다고 말했습니까?
누가 경찰이 기자를 찾았다고 전했습니까?
누가 선생님이 제니를 칭찬했다고 들었니?
누가 큰 형이 꼬마를 놀렸다고 기억했니?
누가 목사가 죄수들을 축복했다고 알게 되었습니까?
누가 에이전트가 여배우를 만졌다고 의심했니?
누가 북한이 남한을 공격했다고 밝혔습니까?
누가 친구들이 선아를 싫어했다고 눈치챘니?
누가 회사가 수미를 스카우트했다고 발표했니?
누가 임원들이 회장을 따돌렸다고 알아냈습니까?
누가 프로듀서가 작가를 야단쳤다고 말했니?
누가 화가가 평론가를 초대했다고 전했습니까?
누가 부모님이 나미를 격려했다고 들었니?
누가 메시가 호날두를 이겼다고 기억했니?
누가 봉사자들이 군인들을 먹였다고 알게 되었니?
누가 베트맨이 수퍼맨을 밀었다고 의심했니?
누가 롬니가 오바마를 모욕했다고 밝혔습니까?
누가 니콜이 현아를 원했다고 눈치챘니?
누가 모네가 고흐를 베꼈다고 발표했습니까?
누가 총리가 의원을 꼬셨다고 알아냈습니까?
누가 선배가 후배를 겁줬다고 말했니?
누가 히틀러가 유대인을 학살했다고 전했습니까?
누가 제니가 에릭을 떠났다고 들었습니까?
누가 박근혜가 문재인을 언급했다고 기억했습니까?
누가 주인공이 악당을 물리쳤다고 알게 되었습니까?
누가 남편이 아내를 폭행했다고 의심했습니까?
누가 노인들이 공화당을 지지했다고 밝혔니?
누가 부사장이 인턴을 눈여겨봤다고 눈치챘니?
누가 사업가가 변호사를 고소했다고 발표했니?
누가 지오가 마리를 이용했다고 알아냈니?

Condition 2: an embedded *wh*-word with a *that*-clause
반장은 수지가 누구를 좋아했다고 말했니?
팅커벨은 피터팬이 누구를 응원했다고 전했니?
준호는 강아지가 누구를 쫓아갔다고 들었니?
기자는 크리스가 누구를 때렸다고 기억했습니까?
수녀는 고아들이 누구를 보살폈다고 알게 되었니?
선생님은 미애가 누구를 속였다고 의심했습니까?
감독은 지니가 누구를 죽였다고 밝혔습니까?
비서는 대통령이 누구를 사랑했다고 눈치챘습니까?
유엔 총장은 유엔이 누구를 파견했다고 발표했습니까?
동생은 오빠가 누구를 꼬집었다고 알아냈니?
할아버지는 할머니가 누구를 걱정했다고 말했습니까?
기자는 경찰이 누구를 찾았다고 전했습니까?
제니는 선생님이 누구를 칭찬했다고 들었니?
꼬마는 큰 형이 누구를 놀렸다고 기억했니?
죄수들은 목사가 누구를 축복했다고 알게 되었습니까?
여배우는 에이전트가 누구를 만졌다고 의심했니?
남한은 북한이 누구를 공격했다고 밝혔습니까?
선아는 친구들이 누구를 싫어했다고 눈치챘니?
수미는 회사가 누구를 스카우트했다고 발표했니?
회장은 임원들이 누구를 따돌렸다고 알아냈습니까?
작가는 프로듀서가 누구를 야단쳤다고 말했니?
평론가는 화가가 누구를 초대했다고 전했습니까?
나미는 부모님이 누구를 격려했다고 들었니?
호날두는 메시가 누구를 이겼다고 기억했니?
군인들은 봉사자들이 누구를 먹였다고 알게 되었니?
수퍼맨은 베트맨이 누구를 밀었다고 의심했니?
오바마는 롬니가 누구를 모욕했다고 밝혔습니까?
현아는 니콜이 누구를 원했다고 눈치챘니?
고흐는 모네가 누구를 베꼈다고 발표했습니까?
의원은 총리가 누구를 꼬셨다고 알아냈습니까?
후배는 선배가 누구를 겁줬다고 말했니?
스탈린은 히틀러가 누구를 학살했다고 전했습니까?
에릭은 제니가 누구를 떠났다고 들었습니까?
문재인은 박근혜가 누구를 언급했다고 기억했습니까?
애인은 주인공이 누구를 물리쳤다고 알게 되었습니까?
아내는 남편이 누구를 폭행했다고 의심했습니까?
공화당은 노인들이 누구를 지지했다고 밝혔니?
인턴은 부사장이 누구를 눈여겨봤다고 눈치챘니?
변호사는 사업가가 누구를 고소했다고 발표했니?
마리는 지오가 누구를 이용했다고 알아냈니?

Condition 3: a matrix *wh*-word with a *wh*-clause
누가 수지가 반장을 좋아했는지 말했니?

누가 피터팬이 팅커벨을 응원했는지 전했니?
누가 강아지가 준호를 쫓아갔는지 들었니?
누가 크리스가 기자를 때렸는지 기억했습니까?
누가 고아들이 장애인들을 보살폈는지 알게 되었니?
누가 미애가 선생님을 속였는지 의심했습니까?
누가 지니가 감독을 죽였는지 밝혔습니까?
누가 대통령이 비서를 사랑했는지 눈치챘습니까?
누가 유엔이 간호사를 파견했는지 발표했습니까?
누가 오빠가 동생을 꼬집었는지 알아냈니?
누가 할머니가 할아버지를 걱정했는지 말했습니까?
누가 경찰이 기자를 찾았는지 전했습니까?
누가 선생님이 제니를 칭찬했는지 들었니?
누가 큰 형이 꼬마를 놀렸는지 기억했니?
누가 목사가 죄수들을 축복했는지 알게 되었습니까?
누가 에이전트가 여배우를 만졌는지 의심했니?
누가 북한이 남한을 공격했는지 밝혔습니까?
누가 친구들이 선아를 싫어했는지 눈치챘니?
누가 회사가 수미를 스카우트했는지 발표했니?
누가 임원들이 회장을 따돌렸는지 알아냈습니까?
누가 프로듀서가 작가를 야단쳤는지 말했니?
누가 화가가 평론가를 초대했는지 전했습니까?
누가 부모님이 나미를 격려했는지 들었니?
누가 메시가 호날두를 이겼는지 기억했니?
누가 봉사자들이 군인들을 먹였는지 알게 되었니?
누가 베트맨이 수퍼맨을 밀었는지 의심했니?
누가 롬니가 오바마를 모욕했는지 밝혔습니까?
누가 니콜이 현아를 원했는지 눈치챘니?
누가 모네가 고흐를 베꼈는지 발표했습니까?
누가 총리가 의원을 꼬셨는지 알아냈습니까?
누가 선배가 후배를 겁줬는지 말했니?
누가 히틀러가 유대인을 학살했는지 전했습니까?
누가 제니가 에릭을 떠났는지 들었습니까?
누가 박근혜가 문재인을 언급했는지 기억했습니까?
누가 주인공이 악당을 물리쳤는지 알게 되었습니까?
누가 남편이 아내를 폭행했는지 의심했습니까?
누가 노인들이 공화당을 지지했는지 밝혔니?
누가 부사장이 인턴을 눈여겨봤는지 눈치챘니?
누가 사업가가 변호사를 고소했는지 발표했니?
누가 지오가 마리를 이용했는지 알아냈니?

Condition 4: an embedded *wh*-word with a *wh*-clause
반장은 수지가 누구를 좋아했는지 말했니?
팅커벨은 피터팬이 누구를 응원했는지 전했니?
준호는 강아지가 누구를 쫓아갔는지 들었니?

기자는 크리스가 누구를 때렸는지 기억했습니까?
수녀는 고아들이 누구를 보살폈는지 알게 되었니?
선생님은 미애가 누구를 속였는지 의심했습니까?
감독은 지니가 누구를 죽였는지 밝혔습니까?
비서는 대통령이 누구를 사랑했는지 눈치챘습니까?
유엔 총장은 유엔이 누구를 파견했는지 발표했습니까?
동생은 오빠가 누구를 꼬집었는지 알아냈니?
할아버지는 할머니가 누구를 걱정했는지 말했습니까?
기자는 경찰이 누구를 찾았는지 전했습니까?
제니는 선생님이 누구를 칭찬했는지 들었니?
꼬마는 큰 형이 누구를 놀렸는지 기억했니?
죄수들은 목사가 누구를 축복했는지 알게 되었습니까?
여배우는 에이전트가 누구를 만졌는지 의심했니?
남한은 북한이 누구를 공격했는지 밝혔습니까?
선아는 친구들이 누구를 싫어했는지 눈치챘니?
수미는 회사가 누구를 스카우트했는지 발표했니?
회장은 임원들이 누구를 따돌렸는지 알아냈습니까?
작가는 프로듀서가 누구를 야단쳤는지 말했니?
평론가는 화가가 누구를 초대했는지 전했습니까?
나미는 부모님이 누구를 격려했는지 들었니?
호날두는 메시가 누구를 이겼는지 기억했니?
군인들은 봉사자들이 누구를 먹였는지 알게 되었니?
수퍼맨은 베트맨이 누구를 밀었는지 의심했니?
오바마는 롬니가 누구를 모욕했는지 밝혔습니까?
현아는 니콜이 누구를 원했는지 눈치챘니?
고흐는 모네가 누구를 베꼈는지 발표했습니까?
의원은 총리가 누구를 꼬셨는지 알아냈습니까?
후배는 선배가 누구를 겁줬는지 말했니?
스탈린은 히틀러가 누구를 학살했는지 전했습니까?
에릭은 제니가 누구를 떠났는지 들었습니까?
문재인은 박근혜가 누구를 언급했는지 기억했습니까?
애인은 주인공이 누구를 물리쳤는지 알게 되었습니까?
아내는 남편이 누구를 폭행했는지 의심했습니까?
공화당은 노인들이 누구를 지지했는지 밝혔니?
인턴은 부사장이 누구를 눈여겨봤는지 눈치챘니?
변호사는 사업가가 누구를 고소했는지 발표했니?
마리는 지오가 누구를 이용했는지 알아냈니?

Experiment 2: canonical adjunct-island

Condition 1: a matrix *wh*-word with a *that*-clause
누가 의사가 병원장을 진료했다고 기록했니?
누가 왕자가 신데렐라를 안았다고 고백했습니까?
누가 과학자가 조교를 혼냈다고 확신했니?

누가 가수가 댄서를 무시했다고 느꼈니?
누가 사장이 회계사를 해고했다고 얘기했습니까?
누가 목사가 거지를 도왔다고 믿었습니까?
누가 축구선수가 발레리나를 귀찮게했다고 생각했니?
누가 장관이 스파이를 환영했다고 주장했습니까?
누가 애플이 삼성을 기만했다고 신고했습니까?
누가 중국인이 일본인을 증오했다고 오해했니?
누가 교수가 미애를 가르쳤다고 거짓말했니?
누가 엄마가 할머니를 버렸다고 고백했습니까?
누가 신랑이 신부를 속였다고 확신했니?
누가 미용사가 매니저를 깔봤다고 느꼈습니까?
누가 요리사가 웨이터를 욕했다고 얘기했습니까?
누가 김태희가 비를 만났다고 믿었니?
누가 고양이가 아이를 깨웠다고 생각했니?
누가 청중이 발표자를 비난했다고 주장했습니까?
누가 사울이 다윗을 모함했다고 신고했습니까?
누가 프랑스가 스파이를 고용했다고 오해했습니까?
누가 간호사가 환자를 치료했다고 거짓말했습니까?
누가 베토벤이 모짜르트를 괴롭혔다고 고백했습니까?
누가 911 이 아들을 구했다고 확신했습니까?
누가 시어머니가 며느리를 째려보았다고 느꼈니?
누가 연예인이 파파라치를 피했다고 얘기했니?
누가 소녀시대가 수퍼주니어를 데려왔다고 믿었니?
누가 음악가가 미술가를 방문했다고 생각했습니까?
누가 테러범이 대통령을 협박했다고 주장했습니까?
누가 소정이가 수애를 살해했다고 신고했습니까?
누가 마술사가 소녀를 유혹했다고 오해했니?
누가 시장이 경찰을 철수했다고 거짓말했습니까?
누가 김하늘이 장동건을 짝사랑했다고 고백했니?
누가 할머니가 손자를 알아봤다고 확신했습니까?
누가 매니저가 팀리더를 편애했다고 느꼈니?
누가 팥쥐가 콩쥐를 미워했다고 얘기했니?
누가 군대가 국민을 보호했다고 믿었습니까?
누가 고모가 택연을 소개했다고 생각했니?
누가 탑이 제시카를 기다렸다고 주장했니?
누가 경찰이 정치가를 풀어줬다고 신고했습니까?
누가 톰이 제리를 흉보았다고 오해했니?

Condition 2: an embedded *wh*-word with a *that*-clause
병원장은 의사가 누구를 진료했다고 기록했니?
신데렐라는 왕자가 누구를 안았다고 고백했습니까?
조교는 과학자가 누구를 혼냈다고 확신했니?
댄서는 가수가 누구를 무시했다고 느꼈니?
회계사는 사장이 누구를 해고했다고 얘기했습니까?

거지는 목사가 누구를 도왔다고 믿었습니까?
발레리나는 축구선수가 누구를 귀찮게했다고 생각했니?
스파이는 장관이 누구를 환영했다고 주장했습니까?
삼성은 애플이 누구를 기만했다고 신고했습니까?
일본인은 중국인이 누구를 증오했다고 오해했니?
미애는 교수가 누구를 가르쳤다고 거짓말했니?
할머니는 엄마가 누구를 버렸다고 고백했습니까?
신부는 신랑이 누구를 속였다고 확신했니?
매니저는 미용사가 누구를 깔봤다고 느꼈습니까?
웨이터는 요리사가 누구를 욕했다고 얘기했습니까?
비는 김태희가 누구를 만났다고 믿었니?
아이는 고양이가 누구를 깨웠다고 생각했니?
발표자는 청중이 누구를 비난했다고주장했습니까?
다윗은 사울이 누구를 모함했다고 신고했습니까?
이란은 프랑스가 누구를 고용했다고 오해했습니까?
환자는 간호사가 누구를 치료했다고 거짓말했습니까?
모짜르트는 베토벤이 누구를 괴롭혔다고 고백했습니까?
아들은 911 이 누구를 구했다고 확신했습니까?
며느리는 시어머니가 누구를 째려보았다고 느꼈니?
파파라치는 연예인이 누구를 피했다고 얘기했니?
수퍼주니어는 소녀시대가 누구를 데려왔다고 믿었니?
미술가는 음악가가 누구를 방문했다고 생각했습니까?
대통령은 테러범이 누구를 협박했다고 주장했습니까?
수애는 소정이가 누구를 살해했다고 신고했습니까?
소녀는 마술사가 누구를 유혹했다고 오해했니?
경찰은 시장이 누구를 철수했다고 거짓말했습니까?
장동건은 김하늘이 누구를 짝사랑했다고 고백했니?
손자는 할머니가 누구를 알아봤다고 확신했습니까?
팀리더는 매니저가 누구를 편애했다고 느꼈니?
콩쥐는 팥쥐가 누구를 미워했다고 얘기했니?
국민은 군대가 누구를 보호했다고 믿었습니까?
택연은 고모가 누구를 소개했다고 생각했니?
제시카는 탑이 누구를 기다렸다고 주장했니?
정치가는 경찰이 누구를 풀어줬다고 신고했습니까?
제리는 톰이 누구를 흉보았다고 오해했니?

Condition 3: a matrix *wh*-word with an adjunct-clause
누가 의사가 병원장을 진료했을 때 떠났니?
누가 왕자가 신데렐라를 안았을 때 울었습니까?
누가 과학자가 조교를 혼냈을 때 당황했니?
누가 가수가 댄서를 무시했을 때 열받았니?
누가 사장이 회계사를 해고했을 때 놀랐습니까?
누가 목사가 거지를 도왔을 때 나타났습니까?
누가 축구선수가 발레리나를 귀찮게했을 때 짜증냈니?

누가 장관이 스파이를 환영했을 때 소리쳤습니까?
누가 애플이 삼성을 기만했을 때 화냈습니까?
누가 중국인이 일본인을 증오했을 때 놀랐니?
누가 교수가 미애를 가르쳤을 때 사라졌니?
누가 엄마가 할머니를 버렸을 때 쓰러졌습니까?
누가 신랑이 신부를 속였을 때 울었니?
누가 미용사가 매니저를 깔봤을 때 화냈습니까?
누가 요리사가 웨이터를 욕했을 때 당황했습니까?
누가 김태희가 비를 만났을 때 나타났니?
누가 고양이가 아이를 깨웠을 때 짜증냈니?
누가 청중이 발표자를 비난했을 때 소리쳤습니까?
누가 사울이 다윗을 모함했을 때 열받았습니까?
누가 프랑스가 스파이를 고용했을 때 실망했습니까?
누가 간호사가 환자를 치료했을 때 쓰러졌습니까?
누가 베토벤이 모짜르트를 괴롭혔을 때 화냈습니까?
누가 911 이 아들을 구했을 때 기뻐했습니까?
누가 시어머니가 며느리를 째려보았을 때 열받았니?
누가 연예인이 파파라치를 피했을 때 실망했니?
누가 소녀시대가 수퍼주니어를 데려왔을 때 나타났니?
누가 음악가가 미술가를 방문했을 때 떠났습니까?
누가 테러범이 대통령을 협박했을 때 소리쳤습니까?
누가 소정이가 수애를 살해했을 때 쓰러졌습니까?
누가 마술사가 소녀를 유혹했을 때 짜증냈니?
누가 시장이 경찰을 철수했을 때 사라졌습니까?
누가 김하늘이 장동건을 짝사랑했을 때 슬퍼했니?
누가 할머니가 손자를 알아봤을 때 기뻐했습니까?
누가 매니저가 팀리더를 편애했을 때 울었니?
누가 팥쥐가 콩쥐를 미워했을 때 화냈니?
누가 군대가 국민을 보호했을 때 기뻐했습니까?
누가 고모가 택연을 소개했을 때 기뻐했니?
누가 탑이 제시카를 기다렸을 때 나타났니?
누가 경찰이 정치가를 풀어줬을 때 소리쳤습니까?
누가 톰이 제리를 흉보았을 때 짜증냈니?

Condition 4: an embedded *wh*-word with an adjunct clause
병원장은 의사가 누구를 진료했을 때 떠났니?
신데렐라는 왕자가 누구를 안았을 때 울었습니까?
조교는 과학자가 누구를 혼냈을 때 당황했니?
댄서는 가수가 누구를 무시했을 때 열받았니?
회계사는 사장이 누구를 해고했을 때 놀랐습니까?
거지는 목사가 누구를 도왔을 때 나타났습니까?
발레리나는 축구선수가 누구를 귀찮게했을 때 짜증냈니?
스파이는 장관이 누구를 환영했을 때 소리쳤습니까?
삼성은 애플이 누구를 기만했을 때 화냈습니까?

일본인은 중국인이 누구를 증오했을 때 놀랐니?
미애는 교수가 누구를 가르쳤을 때 사라졌니?
할머니는 엄마가 누구를 버렸을 때 쓰러졌습니까?
신부는 신랑이 누구를 속였을 때 울었니?
매니저는 미용사가 누구를 깔봤을 때 화냈습니까?
웨이터는 요리사가 누구를 욕했을 때 당황했습니까?
비는 김태희가 누구를 만났을 때 나타났니?
아이는 고양이가 누구를 깨웠을 때 짜증냈니?
발표자는 청중이 누구를 비난했을 때 소리쳤습니까?
다윗은 사울이 누구를 모함했을 때 열받았습니까?
이란은 프랑스가 누구를 고용했을 때 실망했습니까?
환자는 간호사가 누구를 치료했을 때 쓰러졌습니까?
모짜르트는 베토벤이 누구를 괴롭혔을 때 화냈습니까?
아들은 911 이 누구를 구했을 때 기뻐했습니까?
며느리는 시어머니가 누구를 째려보았을 때 열받았니?
파파라치는 연예인이 누구를 피했을 때 실망했니?
수퍼주니어는 소녀시대가 누구를 데려왔을 때 나타났니?
미술가는 음악가가 누구를 방문했을 때 떠났습니까?
대통령은 테러범이 누구를 협박했을 때 소리쳤습니까?
수애는 소정이가 누구를 살해했을 때 쓰러졌습니까?
소녀는 마술사가 누구를 유혹했을 때 짜증냈니?
경찰은 시장이 누구를 철수했을 때 사라졌습니까?
장동건은 김하늘이 누구를 짝사랑했을 때 슬퍼했니?
손자는 할머니가 누구를 알아봤을 때 기뻐했습니까?
팀리더는 매니저가 누구를 편애했을 때 울었니?
콩쥐는 팥쥐가 누구를 미워했을 때 화냈니?
국민은 군대가 누구를 보호했을 때 기뻐했습니까?
택연은 고모가 누구를 소개했을 때 기뻐했니?
제시카는 탑이 누구를 기다렸을 때 나타났니?
정치가는 경찰이 누구를 풀어줬을 때 소리쳤습니까?
제리는 톰이 누구를 흉보았을 때 짜증냈니?

Experiment 3: scrambled *wh*-island

Condition 1: a matrix *wh*-word with a *that*-clause
수지가 반장을 좋아했다고 누가 말했니?
피터팬이 팅커벨을 응원했다고 누가 전했니?
강아지가 준호를 쫓아갔다고 누가 들었니?
크리스가 기자를 때렸다고 누가 기억했습니까?
고아들이 장애인들을 보살폈다고 누가 알게 되었니?
미애가 선생님을 속였다고 누가 의심했습니까?
지니가 감독을 죽였다고 누가 밝혔습니까?
대통령이 비서를 사랑했다고 누가 눈치챘습니까?
유엔이 간호사를 파견했다고 누가 발표했습니까?

오빠가 동생을 꼬집었다고 누가 알아냈니?
할머니가 할아버지를 걱정했다고 누가 말했습니까?
경찰이 기자를 찾았다고 누가 전했습니까?
선생님이 제니를 칭찬했다고 누가 들었니?
큰 형이 꼬마를 놀렸다고 누가 기억했니?
목사가 죄수들을 축복했다고 누가 알게 되었습니까?
에이전트가 여배우를 만졌다고 누가 의심했니?
북한이 남한을 공격했다고 누가 밝혔습니까?
친구들이 선아를 싫어했다고 누가 눈치챘니?
회사가 수미를 스카우트했다고 누가 발표했니?
임원들이 회장을 따돌렸다고 누가 알아냈습니까?
프로듀서가 작가를 야단쳤다고 누가 말했니?
화가가 평론가를 초대했다고 누가 전했습니까?
부모님이 나미를 격려했다고 누가 들었니?
메시가 호날두를 이겼다고 누가 기억했니?
봉사자들이 군인들을 먹였다고 누가 알게 되었니?
베트맨이 수퍼맨을 밀었다고 누가 의심했니?
롬니가 오바마를 모욕했다고 누가 밝혔습니까?
니콜이 현아를 원했다고 누가 눈치챘니?
모네가 고흐를 베꼈다고 누가 발표했습니까?
총리가 의원을 꼬셨다고 누가 알아냈습니까?
선배가 후배를 겁줬다고 누가 말했니?
히틀러가 유대인을 학살했다고 누가 전했습니까?
제니가 에릭을 떠났다고 누가 들었습니까?
박근혜가 문재인을 언급했다고 누가 기억했습니까?
주인공이 악당을 물리쳤다고 누가 알게 되었습니까?
남편이 아내를 폭행했다고 누가 의심했습니까?
노인들이 공화당을 지지했다고 누가 밝혔니?
부사장이 인턴을 눈여겨봤다고 누가 눈치챘니?
사업가가 변호사를 고소했다고 누가 발표했니?
지오가 마리를 이용했다고 누가 알아냈니?

Condition 2: an embedded *wh*-word with a *that*-clause
수지가 누구를 좋아했다고 반장이 말했니?
피터팬이 누구를 응원했다고 팅커벨이 전했니?
강아지가 누구를 쫓아갔다고 준호가 들었니?
크리스가 누구를 때렸다고 기자는 기억했습니까?
고아들이 누구를 보살폈다고 수녀가 알게 되었니?
미애가 누구를 속였다고 선생님이 의심했습니까?
지니가 누구를 죽였다고 감독이 밝혔습니까?
대통령이 누구를 사랑했다고 비서가 눈치챘습니까?
유엔이 누구를 파견했다고 유엔 총장이 발표했습니까?
오빠가 누구를 꼬집었다고 동생이 알아냈니?
할머니가 누구를 걱정했다고 할아버지가 말했습니까?

경찰이 누구를 찾았다고 기자가 전했습니까?
선생님이 누구를 칭찬했다고 제니가 들었니?
큰 형이 누구를 놀렸다고 꼬마가 기억했니?
목사가 누구를 축복했다고 죄수들이 알게 되었습니까?
에이전트가 누구를 만졌다고 여배우가 의심했니?
북한이 누구를 공격했다고 남한이 밝혔습니까?
친구들이 누구를 싫어했다고 선아가 눈치챘니?
회사가 누구를 스카우트했다고 수미가 발표했니?
임원들이 누구를 따돌렸다고 회장이 알아냈습니까?
프로듀서가 누구를 야단쳤다고 작가가 말했니?
화가가 누구를 초대했다고 평론가가 전했습니까?
부모님이 누구를 격려했다고 나미가 들었니?
메시가 누구를 이겼다고 호날두가 기억했니?
봉사자들이 누구를 먹였다고 군인들이 알게 되었니?
베트맨이 누구를 밀었다고 수퍼맨이 의심했니?
롬니가 누구를 모욕했다고 오바마가 밝혔습니까?
니콜이 누구를 원했다고 현아가 눈치챘니?
모네가 누구를 베꼈다고 고흐가 발표했습니까?
총리가 누구를 꼬셨다고 의원이 알아냈습니까?
선배가 누구를 겁줬다고 후배가 말했니?
히틀러가 누구를 학살했다고 스탈린이 전했습니까?
제니가 누구를 떠났다고 에릭이 들었습니까?
박근혜가 누구를 언급했다고 문재인이 기억했습니까?
주인공이 누구를 물리쳤다고 애인이 알게 되었습니까?
남편이 누구를 폭행했다고 아내가 의심했습니까?
노인들이 누구를 지지했다고 공화당이 밝혔니?
부사장이 누구를 눈여겨봤다고 인턴이 눈치챘니?
사업가가 누구를 고소했다고 변호사가 발표했니?
지오가 누구를 이용했다고 마리는 알아냈니?

Condition 3: a matrix *wh*-word with a *wh*-clause
수지가 반장을 좋아했는지 누가 말했니?
피터팬이 팅커벨을 응원했는지 누가 전했니?
강아지가 준호를 쫓아갔는지 누가 들었니?
크리스가 기자를 때렸는지 누가 기억했습니까?
고아들이 장애인들을 보살폈는지 누가 알게 되었니?
미애가 선생님을 속였는지 누가 의심했습니까?
지니가 감독을 죽였는지 누가 밝혔습니까?
대통령이 비서를 사랑했는지 누가 눈치챘습니까?
유엔이 간호사를 파견했는지 누가 발표했습니까?
오빠가 동생을 꼬집었는지 누가 알아냈니?
할머니가 할아버지를 걱정했는지 누가 말했습니까?
경찰이 기자를 찾았는지 누가 전했습니까?

선생님이 제니를 칭찬했는지 누가 들었니?
큰 형이 꼬마를 놀렸는지 누가 기억했니?
목사가 죄수들을 축복했는지 누가 알게 되었습니까?
에이전트가 여배우를 만졌는지 누가 의심했니?
북한이 남한을 공격했는지 누가 밝혔습니까?
친구들이 선아를 싫어했는지 누가 눈치챘니?
회사가 수미를 스카우트했는지 누가 발표했니?
임원들이 회장을 따돌렸는지 누가 알아냈습니까?
프로듀서가 작가를 야단쳤는지 누가 말했니?
화가가 평론가를 초대했는지 누가 전했습니까?
부모님이 나미를 격려했는지 누가 들었니?
메시가 호날두를 이겼는지 누가 기억했니?
봉사자들이 군인들을 먹였는지 누가 알게 되었니?
베트맨이 수퍼맨을 밀었는지 누가 의심했니?
롬니가 오바마를 모욕했는지 누가 밝혔습니까?
니콜이 현아를 원했는지 누가 눈치챘니?
모네가 고흐를 베꼈는지 누가 발표했습니까?
총리가 의원을 꼬셨는지 누가 알아냈습니까?
선배가 후배를 겁줬는지 누가 말했니?
히틀러가 유대인을 학살했는지 누가 전했습니까?
제니가 에릭을 떠났는지 누가 들었습니까?
박근혜가 문재인을 언급했는지 누가 기억했습니까?
주인공이 악당을 물리쳤는지 누가 알게 되었습니까?
남편이 아내를 폭행했는지 누가 의심했습니까?
노인들이 공화당을 지지했는지 누가 밝혔니?
부사장이 인턴을 눈여겨봤는지 누가 눈치챘니?
사업가가 변호사를 고소했는지 누가 발표했니?
지오가 마리를 이용했는지 누가 알아냈니?

Condition 4: an embedded *wh*-word with a *wh*-clause
수지가 누구를 좋아했는지 반장이 말했니?
피터팬이 누구를 응원했는지 팅커벨이 전했니?
강아지가 누구를 쫓아갔는지 준호가 들었니?
크리스가 누구를 때렸는지 기자는 기억했습니까?
고아들이 누구를 보살폈는지 수녀가 알게 되었니?
미애가 누구를 속였는지 선생님이 의심했습니까?
지니가 누구를 죽였는지 감독이 밝혔습니까?
대통령이 누구를 사랑했는지 비서가 눈치챘습니까?
유엔이 누구를 파견했는지 유엔 총장이 발표했습니까?
오빠가 누구를 꼬집었는지 동생이 알아냈니?
할머니가 누구를 걱정했는지 할아버지가 말했습니까?
경찰이 누구를 찾았는지 기자가 전했습니까?
선생님이 누구를 칭찬했는지 제니가 들었니?
큰 형이 누구를 놀렸는지 꼬마가 기억했니?

목사가 누구를 축복했는지 죄수들이 알게 되었습니까?
에이전트가 누구를 만졌는지 여배우가 의심했니?
북한이 누구를 공격했는지 남한이 밝혔습니까?
친구들이 누구를 싫어했는지 선아가 눈치챘니?
회사가 누구를 스카우트했는지 수미가 발표했니?
임원들이 누구를 따돌렸는지 회장이 알아냈습니까?
프로듀서가 누구를 야단쳤는지 작가가 말했니?
화가가 누구를 초대했는지 평론가가 전했습니까?
부모님이 누구를 격려했는지 나미가 들었니?
메시가 누구를 이겼는지 호날두가 기억했니?
봉사자들이 누구를 먹였는지 군인들이 알게 되었니?
베트맨이 누구를 밀었는지 수퍼맨이 의심했니?
롬니가 누구를 모욕했는지 오바마가 밝혔습니까?
니콜이 누구를 원했는지 현아가 눈치챘니?
모네가 누구를 베꼈는지 고흐가 발표했습니까?
총리가 누구를 꼬셨는지 의원이 알아냈습니까?
선배가 누구를 겁줬는지 후배가 말했니?
히틀러가 누구를 학살했는지 스탈린이 전했습니까?
제니가 누구를 떠났는지 에릭이 들었습니까?
박근혜가 누구를 언급했는지 문재인이 기억했습니까?
주인공이 누구를 물리쳤는지 애인이 알게 되었습니까?
남편이 누구를 폭행했는지 아내가 의심했습니까?
노인들이 누구를 지지했는지 공화당이 밝혔니?
부사장이 누구를 눈여겨봤는지 인턴이 눈치챘니?
사업가가 누구를 고소했는지 변호사가 발표했니?
지오가 누구를 이용했는지 마리는 알아냈니?

Experiment 4: scrambled adjunct-island

Condition 1: a matrix *wh*-word with a *that*-clause
의사가 병원장을 진료했다고 누가 기록했니?
왕자가 신데렐라를 안았다고 누가 고백했습니까?
과학자가 조교를 혼냈다고 누가 확신했니?
가수가 댄서를 무시했다고 누가 느꼈니?
사장이 회계사를 해고했다고 누가 얘기했습니까?
목사가 거지를 도왔다고 누가 믿었습니까?
축구선수가 발레리나를 귀찮게했다고 누가 생각했니?
장관이 스파이를 환영했다고 누가 주장했습니까?
애플이 삼성을 기만했다고 누가 신고했습니까?
중국인이 일본인을 증오했다고 누가 오해했니?
교수가 미애를 가르쳤다고 누가 거짓말했니?
엄마가 할머니를 버렸다고 누가 고백했습니까?
신랑이 신부를 속였다고 누가 확신했니?
미용사가 매니저를 깔봤다고 누가 느꼈습니까?

요리사가 웨이터를 욕했다고 누가 얘기했습니까?
김태희가 비를 만났다고 누가 믿었니?
고양이가 아이를 깨웠다고 누가 생각했니?
청중이 발표자를 비난했다고 누가 주장했습니까?
사울이 다윗을 모함했다고 누가 신고했습니까?
프랑스가 스파이를 고용했다고 누가 오해했습니까?
간호사가 환자를 치료했다고 누가 거짓말했습니까?
베토벤이 모짜르트를 괴롭혔다고 누가 고백했습니까?
911 이 아들을 구했다고 누가 확신했습니까?
시어머니가 며느리를 째려보았다고 누가 느꼈니?
연예인이 파파라치를 피했다고 누가 얘기했니?
소녀시대가 수퍼주니어를 데려왔다고 누가 믿었니?
음악가가 미술가를 방문했다고 누가 생각했습니까?
테러범이 대통령을 협박했다고 누가 주장했습니까?
소정이가 수애를 살해했다고 누가 신고했습니까?
마술사가 소녀를 유혹했다고 누가 오해했니?
시장이 경찰을 철수했다고 누가 거짓말했습니까?
김하늘이 장동건을 짝사랑했다고 누가 고백했니?
할머니가 손자를 알아봤다고 누가 확신했습니까?
매니저가 팀리더를 편애했다고 누가 느꼈니?
팥쥐가 콩쥐를 미워했다고 누가 얘기했니?
군대가 국민을 보호했다고 누가 믿었습니까?
고모가 택연을 소개했다고 누가 생각했니?
탑이 제시카를 기다렸다고 누가 주장했니?
경찰이 정치가를 풀어줬다고 누가 신고했습니까?
톰이 제리를 흉보았다고 누가 오해했니?

Condition 2: an embedded *wh*-word with a *that*-clause
의사가 누구를 진료했다고 병원장이 기록했니?
왕자가 누구를 안았다고 신데렐라가 고백했습니까?
과학자가 누구를 혼냈다고 조교가 확신했니?
가수가 누구를 무시했다고 댄서가 느꼈니?
사장이 누구를 해고했다고 회계사가 얘기했습니까?
목사가 누구를 도왔다고 거지가 믿었습니까?
축구선수가 누구를 귀찮게했다고 발레리나가 생각했니?
장관이 누구를 환영했다고 스파이가 주장했습니까?
애플이 누구를 기만했다고 삼성이 신고했습니까?
중국인이 누구를 증오했다고 일본인이 오해했니?
교수가 누구를 가르쳤다고 미애는 거짓말했니?
엄마가 누구를 버렸다고 할머니가 고백했습니까?
신랑이 누구를 속였다고 신부가 확신했니?
미용사가 누구를 깔봤다고 매니저가 느꼈습니까?
요리사가 누구를 욕했다고 웨이터가 얘기했습니까?
김태희가 누구를 만났다고 비가 믿었니?

고양이가 누구를 깨웠다고 아이가 생각했니?
청중이 누구를 비난했다고 발표자가 주장했습니까?
사울이 누구를 모함했다고 다윗이 신고했습니까?
프랑스가 누구를 고용했다고 이란이 오해했습니까?
간호사가 누구를 치료했다고 환자가 거짓말했습니까?
베토벤이 누구를 괴롭혔다고 모짜르트가 고백했습니까?
911 이 누구를 구했다고 아들이 확신했습니까?
시어머니가 누구를 째려보았다고 며느리가 느꼈니?
연예인이 누구를 피했다고 파파라치가 얘기했니?
소녀시대가 누구를 데려왔다고 수퍼주니어가 믿었니?
음악가가 누구를 방문했다고 미술가가 생각했습니까?
테러범이 누구를 협박했다고 대통령이 주장했습니까?
소정이가 누구를 살해했다고 수애가 신고했습니까?
마술사가 누구를 유혹했다고 소녀가 오해했니?
시장이 누구를 철수했다고 경찰이 거짓말했습니까?
김하늘이 누구를 짝사랑했다고 장동건이 고백했니?
할머니가 누구를 알아봤다고 손자가 확신했습니까?
매니저가 누구를 편애했다고 팀리더가 느꼈니?
팥쥐가 누구를 미워했다고 콩쥐가 얘기했니?
군대가 누구를 보호했다고 국민이 믿었습니까?
고모가 누구를 소개했다고 택연이 생각했니?
탑이 누구를 기다렸다고 제시카가 주장했니?
경찰이 누구를 풀어줬다고 정치가가 신고했습니까?
톰이 누구를 흉보았다고 제리가 오해했니?

Condition 3: a matrix *wh*-word with an adjunct clause
의사가 병원장을 진료했을 때 누가 떠났니?
왕자가 신데렐라를 안았을 때 누가 울었습니까?
과학자가 조교를 혼냈을 때 누가 당황했니?
가수가 댄서를 무시했을 때 누가 열받았니?
사장이 회계사를 해고했을 때 누가 놀랐습니까?
목사가 거지를 도왔을 때 누가 나타났습니까?
축구선수가 발레리나를 귀찮게했을 때 누가 짜증냈니?
장관이 스파이를 환영했을 때 누가 소리쳤습니까?
애플이 삼성을 기만했을 때 누가 화냈습니까?
중국인이 일본인을 증오했을 때 누가 놀랐니?
교수가 미애를 가르쳤을 때 누가 사라졌니?
엄마가 할머니를 버렸을 때 누가 쓰러졌습니까?
신랑이 신부를 속였을 때 누가 울었니?
미용사가 매니저를 깔봤을 때 누가 화냈습니까?
요리사가 웨이터를 욕했을 때 누가 당황했습니까?
김태희가 비를 만났을 때 누가 나타났니?
고양이가 아이를 깨웠을 때 누가 짜증냈니?
청중이 발표자를 비난했을 때 누가 소리쳤습니까?

사울이 다윗을 모함했을 때 누가 열받았습니까?
프랑스가 스파이를 고용했을 때 누가 실망했습니까?
간호사가 환자를 치료했을 때 누가 쓰러졌습니까?
베토벤이 모짜르트를 괴롭혔을 때 누가 화냈습니까?
911 이 아들을 구했을 때 누가 기뻐했습니까?
시어머니가 며느리를 째려보았을 때 누가 열받았니?
연예인이 파파라치를 피했을 때 누가 실망했니?
소녀시대가 수퍼주니어를 데려왔을 때 누가 나타났니?
음악가가 미술가를 방문했을 때 누가 떠났습니까?
테러범이 대통령을 협박했을 때 누가 소리쳤습니까?
소정이가 수애를 살해했을 때 누가 쓰러졌습니까?
마술사가 소녀를 유혹했을 때 누가 짜증냈니?
시장이 경찰을 철수했을 때 누가 사라졌습니까?
김하늘이 장동건을 짝사랑했을 때 누가 슬퍼했니?
할머니가 손자를 알아봤을 때 누가 기뻐했습니까?
매니저가 팀리더를 편애했을 때 누가 울었니?
팥쥐가 콩쥐를 미워했을 때 누가 화냈니?
군대가 국민을 보호했을 때 누가 기뻐했습니까?
고모가 택연을 소개했을 때 누가 기뻐했니?
탑이 제시카를 기다렸을 때 누가 나타났니?
경찰이 정치가를 풀어줬을 때 누가 소리쳤습니까?
톰이 제리를 흉보았을 때 누가 짜증냈니?

Condition 4: an embedded *wh*-word with an adjunct clause
의사가 누구를 진료했을 때 병원장이 떠났니?
왕자가 누구를 안았을 때 신데렐라가 울었습니까?
과학자가 누구를 혼냈을 때 조교가 당황했니?
가수가 누구를 무시했을 때 댄서가 열받았니?
사장이 누구를 해고했을 때 회계사가 놀랐습니까?
목사가 누구를 도왔을 때 거지가 나타났습니까?
축구선수가 누구를 귀찮게했을 때 발레리나가 짜증냈니?
장관이 누구를 환영했을 때 스파이가 소리쳤습니까?
애플이 누구를 기만했을 때 삼성이 화냈습니까?
중국인이 누구를 증오했을 때 일본인이 놀랐니?
교수가 누구를 가르쳤을 때 미애가 사라졌니?
엄마가 누구를 버렸을 때 할머니가 쓰러졌습니까?
신랑이 누구를 속였을 때 신부가 울었니?
미용사가 누구를 깔봤을 때 매니저가 화냈습니까?
요리사가 누구를 욕했을 때 웨이터가 당황했습니까?
김태희가 누구를 만났을 때 비가 나타났니?
고양이가 누구를 깨웠을 때 아이가 짜증냈니?
청중이 누구를 비난했을 때 발표자가 소리쳤습니까?
사울이 누구를 모함했을 때 다윗이 열받았습니까?
프랑스가 누구를 고용했을 때 이란이 실망했습니까?

환자는 간호사가 누구를 치료했을 때 쓰러졌습니까?
베토벤이 누구를 괴롭혔을 때 모짜르트가 화냈습니까?
911 이 누구를 구했을 때아들이 기뻐했습니까?
시어머니가 누구를 째려보았을 때 며느리가 열받았니?
연예인이 누구를 피했을 때 파파라치가 실망했니?
소녀시대가 누구를 데려왔을 때 수퍼주니어가 나타났니?
음악가가 누구를 방문했을 때 미술가가 떠났습니까?
테러범이 누구를 협박했을 때 대통령이 소리쳤습니까?
소정이가 누구를 살해했을 때 수애가 쓰러졌습니까?
마술사가 누구를 유혹했을 때 소녀가 짜증냈니?
시장이 누구를 철수했을 때 경찰이 사라졌습니까?
김하늘이 누구를 짝사랑했을 때 장동건이 슬퍼했니?
할머니가 누구를 알아봤을 때 손자가 기뻐했습니까?
매니저가 누구를 편애했을 때 팀리더가 울었니?
팥쥐가 누구를 미워했을 때 콩쥐가 화냈니?
군대가 누구를 보호했을 때 국민이 기뻐했습니까?
고모가 누구를 소개했을 때 택연이 기뻐했니?
탑이 누구를 기다렸을 때 제시카가 나타났니?
경찰이 누구를 풀어줬을 때 정치가가 소리쳤습니까?
톰이 누구를 흉보았을 때 제리가 짜증냈니?
